# Design and synthesis of polycyclic sulfones via Diels–Alder reaction and ring-rearrangement metathesis as key steps

**DOI:** 10.3762/bjoc.11.148

**Published:** 2015-08-06

**Authors:** Sambasivarao Kotha, Rama Gunta

**Affiliations:** 1 Department of Chemistry, Indian Institute of Technology-Bombay, Powai, Mumbai-400 076, India, Fax: 022-25767152

**Keywords:** alkenylation, Diels–Alder reaction, ring-rearrangement metathesis, sulfones

## Abstract

Here, we describe a new and simple synthetic strategy to various polycyclic sulfones via Diels–Alder reaction and ring-rearrangement metathesis (RRM) as the key steps. This approach delivers tri- and tetracyclic sulfones with six (*n* = 1), seven (*n* = 2) or eight-membered (*n* = 3) fused-ring systems containing *trans*-ring junctions unlike the conventional all *cis*-ring junctions generally obtained during the RRM sequence. Interestingly the starting materials used are simple and commercially available.

## Introduction

Sulfones [1–8] are popular building blocks [9] in organic synthesis. They are also useful substrates for the Ramberg–Bäcklund reaction [10] and they can be alkylated via carbanion chemistry. Moreover, they are suitable synthons in Diels–Alder (DA) reactions [11–14]. In view of various applications of sulfone derivatives, we envisioned a new synthetic strategy based on ring-rearrangement metathesis (RRM) as a key step. It is worth mentioning that the RRM strategy [15–23] with a variety of substrates affords intricate products that are inaccessible by conventional retrosynthetic routes. Several bicyclo[2.2.1]heptane systems [24–26] are known to undergo RRM. However, in almost all instances the products produced are *cis*-configured at the ring junctions. The main driving force for the RRM of these systems is the release of ring strain. The configuration is transferred from the starting material to the product. In connection with our interest to design new polycycles by RRM [27–28] as a key step, here we conceive unique examples where *cis* and *trans* ring junctions are produced in the RRM reactions.

## Results and Discussion

### Strategy

**Figure 1:**
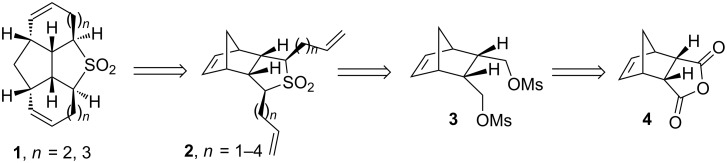
Retrosynthetic approach to polycyclic sulfones.

Our retrosynthetic strategy to diverse sulfone derivatives is shown in Figure 1. The target sulfone derivatives **1** could be synthesized from the functionalized tricyclic sulfone **2** by RRM sequence. The sulfone **2** may be prepared from the dimesylate **3**, which in turn, can be assembled from the known anhydride **4** via reduction followed by mesylation of the resulting diol. Compound **4** could be prepared via DA reaction starting with freshly cracked cyclopentadiene and maleic anhydride (Figure 1).

To realize the strategy shown in Figure 1, we started with the preparation of the known compound **4** [29–30]. Later, the DA adduct **4** was reduced with LiAlH_4_ to deliver the corresponding diol (95%) [31], which was subsequently treated with methanesulfonyl chloride in the presence of triethylamine as a base to obtain the dimesylate **3** (89%). Next, compound **3** was subjected to a cyclization reaction by treating with sodium sulfide nonahydrate (Na_2_S·9H_2_O) using 20% Aliquat^®^ 336 as a phase–transfer catalyst (PTC) to produce the known sulfide **5** (83%) [31].

**Scheme 1:**
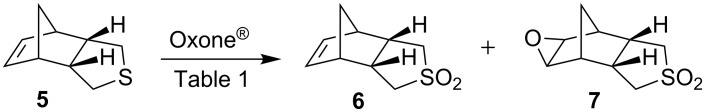
Preparation of the sulfone **6** via oxidation.

Having the sulfide **5** in hand, our next task was to prepare sulfone **6**. In this regard, Trost and Curran [32] have reported the conversion of sulfides to sulfones in the presence of other common functional groups such as olefins by reacting with the oxidizing agent, potassium hydrogen persulfate (KHSO_5_, commercially available as Oxone^®^) in aqueous methanol. Equipped with this information, oxidation of compound **5** was attempted under similar reaction conditions to get the desired sulfone **6** [33] (Scheme 1, Table 1).

**Table 1:** Different reaction conditions used to improve the yield of the sulfone **6**.

Entry	Reaction conditions	**6** yield [%]	**7** yield [%]

1	Oxone^®^ (3 equiv), MeOH, H_2_O, 0 °C, 22 h	29	40
**2**	**Oxone^®^ (2.5 equiv), MeOH, H_2_O, −5 °C, 6 h**	**89**	**8**
3	Oxone^®^ (2.5 equiv), MeOH, H_2_O, −5 °C, 5.5 h	83	15
4	Oxone^®^ (2.2 equiv), MeOH, H_2_O, −8 °C, 4.5 h	82	5
5	Oxone^®^ (2 equiv), MeOH, H_2_O, −20 °C, 5 h	71	5

Initially, when the reaction was carried out at 0 °C, the epoxy sulfone **7** was the major product (Table 1, entry 1). However, after a considerable amount of experimentation (Table 1), the desired sulfone **6** has been produced in 89% yield (Table 1, entry 2) but it was not possible to eliminate the formation of the epoxy sulfone **7**.

**Scheme 2:**
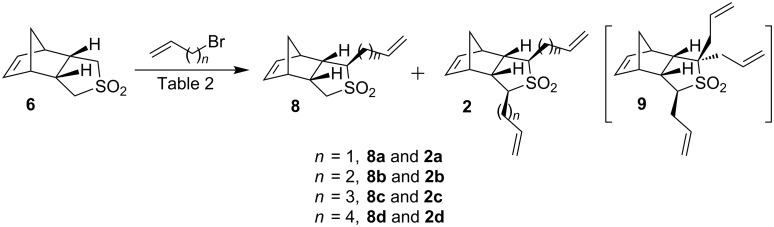
Synthesis of alkenylated sulfone derivatives.

Next, our efforts were directed towards the synthesis of various alkenylated sulfone derivatives. In this regard, Bloch and co-workers reported a useful preparation of monoallylated sulfone **8a** [34]. To this end, we carried out the allylation of sulfone **6** with allyl bromide (1.2 equiv) and *n*-BuLi (2.7 equiv) at −75 °C to rt. The monoallylated sulfone **8a** was obtained in 22% yield and the diallylated sulfone **2a** in 5% yield. Also, 25% of the starting material was recovered. To optimize the yield of diallylated sulfone **2a** various conditions were studied (e.g., NaH and LDA). In this regard, increasing the equivalents of allyl bromide and *n*-BuLi produced the diallylated sulfone **2a** in 80% yield and the monoallylated compound **8a** in 10% yield (Table 2, entry 1a) [35] along with a minor amount (3%) of triallylated sulfone **9** (Scheme 2). However, with an excess amount of base (5 equiv) and allyl bromide the diallylated sulfone **2a** was isolated as a major product and the triallylated sulfone **9** in 6% yield (Table 2, entry 1b). Later, the monoallylated sulfone **8a** has been converted to the desired diallyl compound **2a** (88%) under similar reaction conditions. The structures of the diallyl (**2a**) and triallyl (**9**) sulfones have been confirmed by ^1^H and ^13^C NMR spectral data and further supported by HRMS data. In addition, the structure and stereochemistry of the allyl groups present in compound **2a** have been confirmed by single-crystal X-ray diffraction studies and this data clearly indicated that the allylation had occurred at α-position of the sulfone moiety and the two allyl groups are in *cis*-arrangement with each other [35–37].

**Table 2:** Optimized reaction conditions to realize mono and dialkenylated sulfones.

Entry	*n*	Reaction conditions	Monoalkenylated product yield [%]	Dialkenylated product yield [%]

1a	1	allyl bromide (3 equiv), *n*-BuLi THF, −75 °C to rt, 25 h	**8a** (10)	**2a** (80) & **9**^a^ (3)
1b		allyl bromide (10 equiv), *n*-BuLi THF, −58 °C to rt, 26 h	**8a** (0)	**2a** (80) & **9**^a^ (6)
2	2	4-bromo-1-butene (3 equiv), *n*-BuLi HMPA, THF, −74 °C to rt, 20 h	**8b** (75^b^)	**2b** (21^b^)
3	3	5-bromo-1-pentene (2.5 equiv), *n*-BuLi HMPA, THF, −78 °C to rt, 17.5 h	**8c** (5)	**2c** (57)
4	4	6-bromo-1-hexene (2.8 equiv), *n*-BuLi HMPA, THF, −78 °C to rt, 17 h	**8d** (9)	**2d** (75)

^a^Triallylated product, ^b^isolated yield based on starting material recovered.

Analogously, the alkenylation of sulfone **6** was optimized with other electrophiles and the results are summarized in Table 2 (entries 2–4). In this regard, sulfone **6** was butenylated with 4-bromo-1-butene and *n*-BuLi in the presence of HMPA at −74 °C to rt to deliver the monobutenylated sulfone **8b** in 75% yield. Surprisingly, here a minor amount of the desired dibutenylated sulfone **2b** (21%) was isolated (Table 2, entry 2). However, the monobutenylated sulfone **8b** can be converted to the dibutenylated sulfone **2b** under similar conditions. Next, the same synthetic sequence has been extended to the dipentenyl and the dihexenyl sulfone derivatives. Thus, treatment of sulfone **6** with 5-bromo-1-pentene and *n*-BuLi using HMPA at −78 °C to rt (Table 2, entry 3) gave the desired dipentenylated sulfone **2c** (57%) and a minor amount of monopentenylated sulfone **8c** (5%).

Similarly, we synthesized the hexenyl sulfone derivatives **8d** and **2d** by treating compound **6** with 6-bromo-1-hexene using HMPA and *n*-BuLi at −78 °C. The desired dihexenylated sulfone **2d** has been furnished in 75% yield along with monohexenyl sulfone derivative **8d** (9%, Table 2, entry 4). Based on these optimization studies, it was concluded that it is necessary to use the appropriate number of equivalents of the alkenyl bromide and the suitable base to generate the dialkenylated products (Table 2 and Scheme 2).

**Scheme 3:**
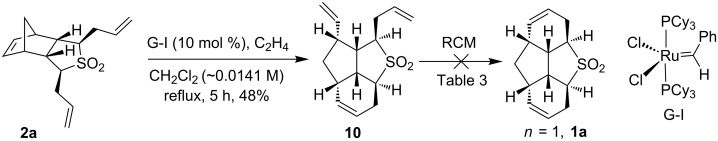
Synthesis of **10** by RRM of **2a**.

After the successful synthesis of various dialkenyl sulfone derivatives **2a–d**, we focussed our attention towards the RRM step. Initially, the diallyl sulfone **2a** (~0.0141 M solution in dry CH_2_Cl_2_) was subjected to RRM using G-I catalyst in the presence of ethylene gas in refluxing CH_2_Cl_2_ to get the tetracyclic sulfone **1a**, however, we isolated the tricyclic sulfone **10** in 48% yield. When the G-I catalyst was replaced with G-II a complex mixture of products was observed as indicated by ^1^H and ^13^C NMR spectral data. Later, compound **10** was treated with conventional Grubbs catalysts under different reaction conditions (Table 3) to obtain the RRM product **1a** (Scheme 3). Unfortunately, the expected compound **1a** was not obtained. The strain present in the *trans*-fused compound **1a** may be responsible for its absence in the RRM sequence.

**Table 3:** Toluene (~0.004 M) reflux conditions to convert **10** to **1a**.

Entry	Conditions	Result

1	G-I (10 mol %), C_2_H_4_, 19 h	SM^a^ recovered
2	G-II (10 mol %), Ti(OiPr)_4_, C_2_H_4_, 24 h	No product^b^
3	HG-II^c^ (10 mol %), Ti(OiPr)_4_, C_2_H_4_, 24 h	No product^b^

^a^Starting material. ^b^SM not recovered, ^c^Hoveyda–Blechert–Grubbs catalyst.

**Scheme 4:**
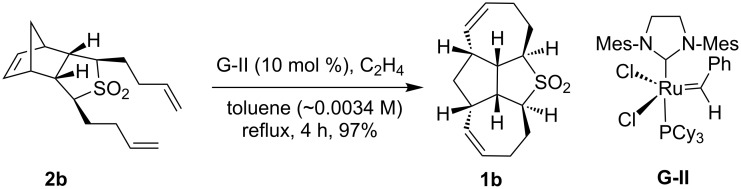
Synthesis of **1b** using RRM.

Interestingly, dibutenyl sulfone **2b** (~0.0034 M solution in toluene) smoothly underwent RRM with Grubbs 2^nd^ generation (G-II) catalyst in the presence of ethylene in refluxing toluene to produce the anticipated tetracyclic sulfone **1b** (97%) (Scheme 4). The sulfone **1b** has been characterized by ^1^H and ^13^C NMR and DEPT-135 spectral data including HRMS data.

**Scheme 5:**
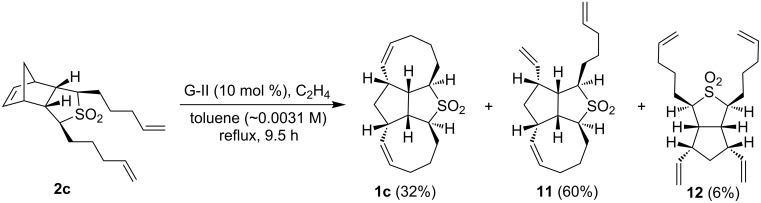
RRM of the dipentenyl sulfone **2c**.

Next, the RRM of dipentenyl sulfone **2c** (~0.0031 M solution in toluene) was carried out under similar reaction conditions to furnish **1c**. Interestingly, the tricyclic sulfone **11** was isolated in 60% along with the expected tetracyclic sulfone **1c** (32%) and a minor amount of ring-opened product **12** (6%, Scheme 5). A complex mixture of products was obtained when compound **2c** was exposed to the metathesis catalyst for a longer period of time as indicated by ^1^H and ^13^C NMR spectral data.

**Scheme 6:**
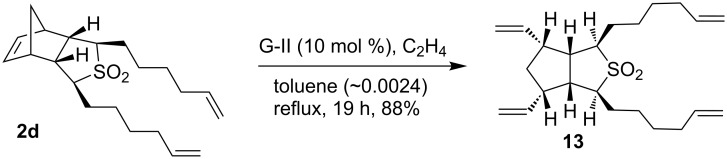
RRM of the dihexenyl sulfone **2d**.

Analogously, dihexenyl sulfone **2d** (~0.0024 M solution in toluene) was treated with G-II catalyst to deliver the RRM product in the presence of ethylene in refluxing toluene. In this regard, only ring-opened sulfone **13** was produced in 88% yield (Scheme 6) and no cyclized product was observed. Presumably, this observation may be explained on the basis that the nine-membered ring product was not formed due to the unfavourable steric interactions involved.

## Conclusion

Several interesting polycyclic sulfone derivatives were designed and assembled involving RRM. The RRM outcome of various sulfones (**2a–d**) depends on the length of the alkenyl chain. In this context, the dibutenyl sulfone derivative **2b** is the most-promising candidate for the RRM protocol. In other instances, for example with propenyl analogue **2a** the partial ring-closing product **10** was obtained. With substrate **2c**, the eight-membered RRM compound **1c** was formed as a minor product and partial ring-closing compound **11** as a major product. With substrate **2d**, only ring-opened product **13** was produced. Interestingly, we demonstrated *trans*-ring junction products are possible in the RRM protocol. It is clear that RRM has a unique place in olefin metathesis [38–45] and further interesting examples are expected in future.

## Supporting Information

File 1 Detailed experimental procedures, characterization data and copies of ^1^H,^13^C NMR and HRMS spectra for all new compounds.
