# Supplementary material for: Design and synthesis of polycyclic sulfones via Diels–Alder reaction and ring-rearrangement metathesis as key steps
Source: Beilstein J Org Chem. 2015 Aug 6;11:1373–8. doi: 10.3762/bjoc.11.148 (PMC4578373; doi:10.3762/bjoc.11.148)
Supplement: File 1 — Detailed experimental procedures, characterization data and copies of 1H,13C NMR and HRMS spectra for all new compounds. [file Beilstein_J_Org_Chem-11-1373-s001.pdf]

Supporting Information

for

Design and synthesis of polycyclic sulfones via Diels–Alder reaction and ring-rearrangement metathesis as key steps

Sambasivarao Kotha* and Rama Gunta

Address: Department of Chemistry, Indian Institute of Technology-Bombay, Powai, Mumbai, India, Fax: 022-25767152

Email: Sambasivarao Kotha* - srk@chem.iitb.ac.in

*Corresponding author

Detailed experimental procedures, characterization data and copies of ^{1}H, ^{13}C NMR and HRMS spectra for all new compounds

Contents

Experimental procedures and characterization data S2

References S13

Copies of ^{1}H, ^{13}C NMR and HRMS spectra of the compounds 2a–d, 8b–d, 1b,c and 10–13; DEPT-135 spectra of the selected compounds S14

Experimental procedures and characterization data

General methods

All the reactions were monitored by employing thin-layer chromatography (TLC) using appropriate mixtures of EtOAc and petroleum ether as mobile phase. Reactions involving oxygen sensitive reagents or catalysts were performed in degassed solvents. Transfer of moisture sensitive materials were carried out using standard syringe–septum techniques and the reactions were maintained under nitrogen or argon atmosphere until work up. Dry dichloromethane (CH_2Cl_2) and toluene were obtained by distillation over P_2O_5, dry hexamethylphosphoramide (HMPA) was prepared by stirring over CaH_2 for 1 h followed by vacuum distillation and anhydrous tetrahydrofuran (THF) was obtained by distillation over sodium benzophenone freshly prior to use. Sodium sulfide nonahydrate ($Na_2S{\cdot}9H_2O$) and *n*-BuLi (1.6 M solution in hexane) were purchased from Acros Organics. Potassium hydrogen persulfate ($KHSO_5$) commercially available as Oxone®. The composition of the Oxone® is $2KHSO_5{\cdot}KHSO_4{\cdot}K_2SO_4$. All the commercial grade reagents were used without further purification.

Techniques

Melting points were recorded on a Veego melting point apparatus and are uncorrected. Nuclear Magnetic Resonance (NMR) spectra were generally recorded on Bruker (Avance™ 400 or Avance™ III 500) spectrometers operated at 400 or 500 MHz for ^{1}H and 100.6 or 125.7 MHz for ^{13}C nuclei. NMR samples were generally made in chloroform-*d* solvent and chemical shifts (δ values) were reported in parts per million (ppm) using tetramethylsilane (TMS) as an internal standard. Coupling constants (*J* values) were reported in hertz (Hz). The high-resolution mass spectrometric (HRMS) measurements were carried out using a Bruker (Maxis Impact) or Micromass Q-ToF spectrometer. Infrared (IR) spectra were recorded on a Nicolet Impact-400 FT IR spectrometer.

Preparation of the sulfide 5

The known sulfide **5** was prepared starting with the anhydride **4** in three steps by following the literature procedure [1] as shown in Scheme S1.

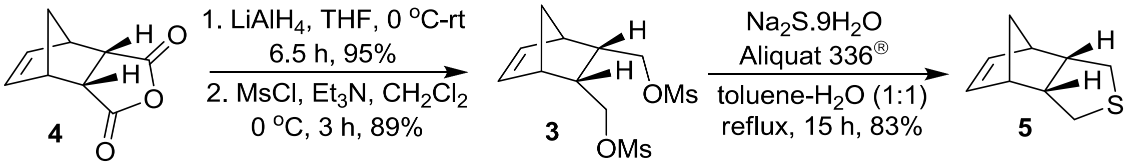

Scheme S1: Preparation of the sulfide **5** starting from the anhydride **4**.

Preparation of the sulfone 6 [2]

To a stirred solution of sulfide **5** (220 mg, 1.44 mmol) in MeOH (8 mL), a solution of Oxone® (1.10 g, 3.61 mmol) in water (8 mL) was added at −5 °C. Then, milky white suspension was formed. This suspension was stirred at the same temperature for 6 h. After completion of the reaction, the solvent was removed under reduced pressure. To the resulting residue water (30 mL) was added and extracted with $CHCl_3$ (3 × 30 mL). The combined organic layers were washed with brine (2 × 20 mL), dried over anhydrous Na_2SO_4 and concentrated in vacuo. The crude product was purified by silica gel column chromatography (30% EtOAc–petroleum ether) to furnish the known sulfone **6** (266 mg, 89%) as colourless flakes and a minor amount of epoxy sulfone **7** (23 mg, 8%) as a white solid.

Sulfone 6: mp: 126–128 °C (lit. [3] mp: 124–126 °C).

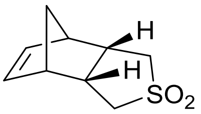

^{1}H NMR (500 MHz, $CDCl_3$): δ (ppm) = 6.26 (t, J = 2.0 Hz, 2H), 3.16–3.12 (m, 2H), 3.28 (br s, 2H), 2.96–2.91 (m, 2H), 2.30–2.25 (m, 2H), 1.75 (dt, J = 9.0, 2.0 Hz, 1H), 1.48 (d, J = 2.0 Hz, 1H); ^{13}C NMR (100.6 MHz, $CDCl_3$): δ (ppm) = 136.9, 53.1, 49.5, 45.1, 39.5; HRMS (ESI, Q-ToF) m/z: calculated for $C_9H_{12}NaO_2S$ $[M+Na]^+$ 207.0450, found: 207.0449.

Epoxy sulfone 7: mp: 238–240 °C (lit. [4] mp: 241–242 °C).

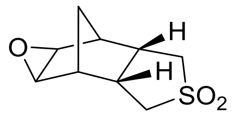

^{1}H NMR (400 MHz, $CDCl_3$): δ (ppm) = 3.28 (br s, 2H), 3.14–3.08 (m, 2H), 2.90–2.83 (m, 4H), 2.73 (br s, 2H), 1.62–1.56 (m, 1H), 0.93 (d, J = 10.8 Hz, 1H); ^{13}C NMR (100.6 MHz, $CDCl_3$): δ (ppm) = 49.0, 48.9, 38.8, 38.3, 28.0.

Preparation of the diallyl sulfone 2a

To a stirred solution of sulfone **6** (153 mg, 0.83 mmol) in anhydrous THF (15 mL) was added *n*-BuLi (2.6 mL, 5 equiv) and allyl bromide (0.83 mL, 9.77 mmol) at −58 °C under nitrogen. The resulting reaction mixture was stirred at rt for 26 h. After completion of the reaction (TLC monitoring), the reaction mixture was quenched with water (5 mL) and the solvent was removed in a rotary evaporator in vacuo. The resulting residue was extracted with diethyl ether (3 × 20 mL). The combined organic layers were washed with brine (2 × 20 mL), dried over anhydrous Na_2SO_4 and concentrated. The crude product was purified by silica gel column chromatography (10% EtOAc–petroleum ether) to get the diallyl sulfone **2a** (176 mg, 80%) as a white crystalline solid and a minor amount of the triallyl sulfone **9** (16 mg, 6%) was obtained as a white solid.

Conversion of the monoallyl sulfone 8a to the diallyl sulfone 2a

To a stirred solution of monoallyl sulfone **8a** (100 mg, 0.44 mmol) in anhydrous THF (5 mL) was added *n*-BuLi (0.33 mL, 1.2 equiv) and allyl bromide (0.04 mL, 0.49 mmol) at −78 °C under nitrogen. After 2 h, the reaction mixture was allowed to reach the rt and stirred further for 23 h. After completion of the reaction, the reaction mixture was quenched with water (2 mL) and the solvent was evaporated under reduced pressure. The resulting residue was extracted with diethyl ether (3 × 30 mL). The combined organic layers were washed with brine (2 × 10 mL) and dried over anhydrous Na_2SO_4. The solvent was removed in vacuo and the crude product was purified by silica gel column chromatography (10% EtOAc–petroleum

ether) to give the desired diallyl sulfone **2a** [80 mg, 88% (based on the starting material, 23 mg recovered)] as a white crystalline solid.

Monoallyl sulfone 8a [5]: $\boldsymbol{R_f}$ = 0.53 (20% EtOAc–petroleum ether).

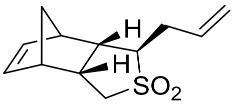

^{1}H NMR (400 MHz, $CDCl_3$): δ (ppm) = 6.23 (t, *J* = 1.1 Hz, 2H), 5.90–5.79 (m, 1H), 5.24 (dq, *J* = 17.0, 1.5 Hz, 1H), 5.17 (dq, *J* = 10.0, 1.2 Hz, 1H), 3.19 (dd, *J* = 13.4, 8.4 Hz, 1H), 3.17 (d, *J* = 1.4 Hz, 2H), 2.83–2.69 (m, 2H), 2.49–2.38 (m, 2H), 2.34–2.25 (m, 2H), 1.72 (dt, *J* = 8.8, 1.9 Hz, 1H), 1.44 (dd, *J* = 8.8, 1.3 Hz, 1H); ^{13}C NMR (125.7 MHz, $CDCl_3$): δ (ppm) = 137.1, 136.6, 133.4, 118.5, 62.4, 52.9, 49.7, 46.6, 45.4, 45.2, 37.8, 30.5; HRMS (ESI, Q-ToF) *m/z*: calculated for $C_{12}H_{16}NaO_2S$ $[M+Na]^+$ 247.0763, found: 247.0764.

Diallyl sulfone 2a [6]: $\boldsymbol{R_f}$ = 0.91 (20% EtOAc–petroleum ether); mp: 75–76 °C.

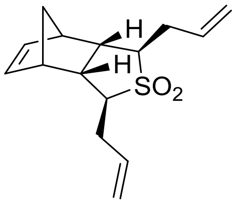

^{1}H NMR (500 MHz, $CDCl_3$): δ (ppm) = 6.20 (br s, 2H), 5.88–5.80 (m, 2H), 5.23 (dd, *J* = 16.9, 1.2 Hz, 2H), 5.16 (d, *J* = 10.0 Hz, 2H), 2.99 (br s, 2H), 2.77–2.72 (m, 2H), 2.45–2.40 (m, 2H), 2.37–2.30 (m, 4H), 1.67 (d, *J* = 8.8 Hz, 1H), 1.39 (d, *J* = 8.8 Hz, 1H); ^{13}C NMR (125.7 MHz, $CDCl_3$): δ (ppm) = 136.9, 133.6, 118.6, 62.5, 49.8, 45.6, 45.3, 30.8; HRMS (ESI, Q-ToF) *m/z*: calculated for $C_{15}H_{20}NaO_2S$ $[M+Na]^+$ 287.1076, found: 287.1077; IR (neat): υ_{max} = 3021, 2978, 1640, 1443, 1306, 1216, 1123 cm^{-1}.

Triallyl sulfone 9: $\boldsymbol{R_f}$ = 0.99 (20% EtOAc–petroleum ether); mp: 75–77 °C.

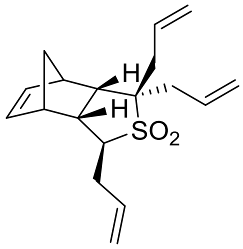

^{1}H NMR (400 MHz, $CDCl_3$): δ (ppm) = 6.23 (dd, *J* = 5.7, 2.8 Hz, 1H), 6.10–6.00 (m, 2H), 5.90–5.80 (m, 2H), 5.26–5.13 (m, 6H), 3.00 (br s, 2H), 2.76–2.68 (m, 2H), 2.62–2.41 (m, 6H), 2.12 (dd, *J* = 15.4, 8.0 Hz, 1H), 1.66 (dt, *J* = 8.6, 1.8 Hz, 1H), 1.41 (d, *J* = 8.6 Hz, 1H); ^{13}C NMR (125.7 MHz, $CDCl_3$): δ (ppm) = 137.9, 135.1, 133.7, 132.7, 131.6, 120.2, 119.5, 118.5, 65.0, 59.7, 52.0, 50.3, 46.6,

45.3, 45.1, 38.1, 36.1, 30.7; HRMS (ESI, Q-ToF) *m/z*: calculated for $C_{18}H_{24}NaO_2S$ $[M+Na]^+$ 327.1389, found: 327.1387; IR (neat): υ_{max} = 3082, 2978, 1637, 1437, 1305, 1123 cm^{-1}.

Synthesis of the butenyl sulfones 8b and 2b

To a solution of sulfone **6** (200 mg, 1.08 mmol) in anhydrous THF (15 mL), was added *n*-BuLi (1.60 mL, 2.4 equiv) at −74 °C and stirred for 40 min. Next, dry HMPA (0.38 mL, 2.17 mmol) followed by 4-bromo-1-butene (0.33 mL, 3.26 mmol) were added and the stirring was continued at the same temperature for 1 h. Later, the reaction mixture was allowed to attain the rt and stirred for another 19 h. After completion of the reaction, the reaction mixture was quenched with water (2 mL) and concentrated under reduced pressure. The resulting residue was extracted with diethyl ether (3 × 20 mL). The combined organic layers were washed with brine (2 × 15 mL), dried over anhydrous Na_2SO_4 and concentrated. Subsequently, the crude product was purified on silica gel column chromatography (8% EtOAc–petroleum ether) to obtain the dibutenyl sulfone **2b** [43 mg, 21% (based on the starting material, 72 mg recovered)] as a colourless liquid and the monobutenyl sulfone **8b** [125 mg, 75% (based on the starting material, 72 mg recovered)] was delivered by eluting the column with 14% EtOAc–petroleum ether as a yellow liquid.

Monobutenyl sulfone 8b: ***R*** R_f = 0.36 (20% EtOAc–petroleum ether).

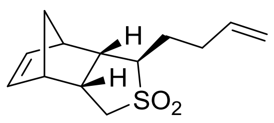

^{1}H NMR (500 MHz, $CDCl_3$): δ (ppm) = 6.23 (t, *J* = 1.8 Hz, 2H), 5.83–5.75 (m, 1H), 5.11 (dq, *J* = 17.1, 1.5 Hz, 1H), 5.06 (dd, *J* = 10.2, 1.4 Hz, 1H), 3.18 (dd, *J* = 13.4, 8.4 Hz, 1H), 3.02 (m, 2H), 2.83–2.76 (m, 1H), 2.44 (td, *J* = 10.0, 3.6 Hz, 1H), 2.38–2.26 (m, 4H), 2.12–2.05 (m, 1H), 1.83–1.76 (m, 1H), 1.73 (dt, *J* = 8.8, 1.8 Hz, 1H), 1.45 (d, *J* = 8.8 Hz, 1H); ^{13}C NMR (125.7 MHz, $CDCl_3$): δ (ppm) = 137.2, 137.0, 136.6, 116.3, 62.0, 53,1, 49.7, 47.0, 45.5, 45.2, 37.9, 30.8, 25.5; HRMS (ESI, Q-ToF) *m/z*: calculated for $C_{13}H_{18}NaO_2S$ $[M+Na]^+$ 261.0920, found: 261.0921; IR (neat): υ_{max} = 3058, 2969, 1641, 1444, 1303, 1138, 914 cm^{-1}.

Dibutenyl sulfone 2b: $\boldsymbol{R_f}$ = 0.64 (20% EtOAc–petroleum ether).

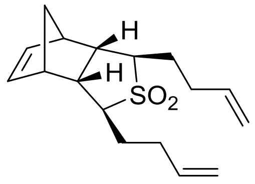

^{1}H NMR (400 MHz, $CDCl_3$): δ (ppm) = 6.19 (t, J = 1.8 Hz, 2H), 5.84–5.73 (m, 2H), 5.13–5.04 (m, 4H), 3.01 (br s, 2H), 2.37–2.25 (m, 8H), 2.14–2.07 (m, 2H), 1.83–1.76 (m, 2H), 1.69 (dt, J = 3.6, 1.8 Hz, 1H), 1.40 (d, J = 8.8 Hz, 1H); ^{13}C NMR (125.7 MHz, $CDCl_3$): δ (ppm) = 137.1, 136.9, 116.3, 62.1, 49.8, 45.5, 45.5, 30.9, 25.7; HRMS (ESI, Q-ToF) *m/z*: calculated for $C_{17}H_{24}NaO_2S$ $[M+Na]^+$ 315.1389, found: 315.1389; IR (neat): υ_{max} = 3068, 2976, 1642, 1453, 1298, 1134, 915 cm^{-1}.

Synthesis of the pentenyl sulfones 8c and 2c

To a solution of sulfone **6** (200 mg, 1.08 mmol) in anhydrous THF (8 mL), was added *n*-BuLi (1.50 mL, 2.2 equiv) and dry HMPA (0.38 mL, 2.17 mmol) followed by 5-bromo-1-pentene (0.32 mL, 2.71 mmol) at −78 °C. The resulting reaction mixture was stirred at the same temperature for 2 h. Next, the reaction mixture was stirred at rt for 17.5 h. After completion of the reaction, the reaction mixture was quenched with water (2 mL) concentrated in vacuo. The subsequent residue was extracted with diethyl ether (3 × 20 mL), combined organic layers were washed with brine (2 × 15 mL) and dried over anhydrous Na_2SO_4. Evaporation of the solvent under reduced pressure gave the crude product which was subjected to silica gel column chromatography (9% EtOAc–petroleum ether) to give the dipentenyl sulfone **2c** (200 mg, 57%) as a colourless liquid. Further elution of the column with 12% EtOAc–petroleum ether, the monopentenyl sulfone **8c** (13 mg, 5%) was obtained as a pale yellow liquid.

Monopentenyl sulfone 8c: $\boldsymbol{R_f}$ = 0.33 (20% EtOAc–petroleum ether).

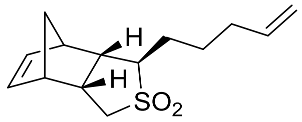

^{1}H NMR (400 MHz, $CDCl_3$): δ (ppm) = 6.27–6.23 (m, 2H), 5.85–5.74 (m, 1H), 5.07–4.98 (m, 2H), 3.18 (dd, J = 13.3, 8.4 Hz, 1H), 3.01 (d, J =1.2 Hz, 2H), 2.78 (qd, J = 10.0, 3.1 Hz, 1H), 2.78 (td, J = 10.0, 3.6 Hz, 1H), 2.31–2.23 (m, 2H), 2.17–2.09 (m, 2H), 2.00–1.92 (m, 2H), 1.76–1.56 (m, 3H), 1.44 (d, J = 8.8 Hz, 1H); ^{13}C NMR (125.7 MHz, $CDCl_3$): δ (ppm) = 137.9, 137.1, 136.8, 115.6, 62.8, 53,1, 49.8,

47.0, 45.6, 45.2, 37.9, 33.8, 26.3, 25.8; HRMS (ESI, Q-ToF) *m/z*: calculated for $C_{14}H_{20}NaO_2S$ $[M+Na]^+$ 275.1076, found: 275.1076; IR (neat): υ_{max} = 3058, 2936, 1641, 1456, 1299, 1137, 914 cm^{-1}.

Dipentenyl sulfone 2c: $\boldsymbol{R_f}$ = 0.63 (20% EtOAc–petroleum ether).

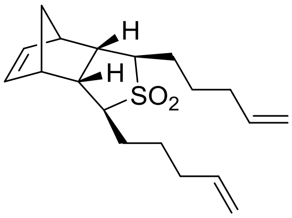

^{1}H NMR (500 MHz, $CDCl_3$): δ (ppm) = 6.23 (t, J = 1.8 Hz, 2H), 5.84–5.76 (m, 2H), 5.04 (dq, J = 17.1, 1.5 Hz, 2H), 5.00–4.98 (m, 2H), 3.00 (s, 2H), 2.33–2.26 (m, 4H), 2.12 (q, J = 7.4 Hz, 4H), 2.00–1.94 (m, 2H), 1.74–1.68 (m, 3H), 1.66–1.60 (m, 4H), 1.41 (d, J = 8.7 Hz, 1H); ^{13}C NMR (125.7 MHz, $CDCl_3$): δ (ppm) = 137.9, 136.9, 115.5, 62.9, 49.9, 45.6, 45.6, 33.8, 26.4, 26.0; HRMS (ESI, Q-ToF) *m/z*: calculated for $C_{19}H_{28}NaO_2S$ $[M+Na]^+$ 343.1702, found: 343.1702; IR (neat): υ_{max} = 3075, 2937, 1640, 1458, 1295, 1133, 912 cm^{-1}.

Synthesis of the hexenyl sulfones 8d and 2d

To a solution of sulfone **6** (500 mg, 2.71 mmol) in anhydrous THF (25 mL), was added *n*-BuLi (4.10 mL, 2.4 equiv) and dry HMPA (0.94 mL, 5.43 mmol) followed by 6-bromo-1-hexene (1.01 mL, 7.60 mmol) at −78 °C under nitrogen. The resulting reaction mixture was stirred at the same temperature for 2 h. Next, the stirring was continued at rt for 15 h. After completion of the reaction, the reaction mixture was quenched with water (5 mL) and the solvent was removed under reduced pressure. The residue was extracted with diethyl ether (3 × 30 mL), the combined organic layers were washed with brine (2 × 20 mL) and dried over anhydrous Na_2SO_4. The solvent was evaporated in vacuo and purification of the crude product on silica gel column chromatography (7% EtOAc–petroleum ether) provided the dihexenyl sulfone **2d** (706 mg, 75%) as a colourless liquid and further elution with 10% EtOAc–petroleum ether gave the monohexenyl sulfone **8d** (64 mg, 9%) as a pale yellow liquid.

Monohexenyl sulfone 8d: $\boldsymbol{R_f}$ = 0.49 (20% EtOAc–petroleum ether).

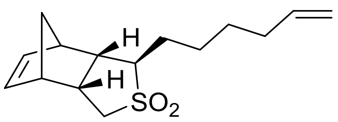

^{1}H NMR (500 MHz, $CDCl_3$): δ (ppm) = 6.26–6.23 (m, 2H), 5.84–5.76 (m, 1H), 5.01 (dq, J = 17.1, 1.6 Hz, 1H), 4.98–4.95 (m, 1H), 3.17 (dd, J = 13.3, 8.4 Hz, 1H), 3.01 (br s, 2H), 2.80–2.75 (m, 1H), 2.43 (td, J = 10.0, 3.5 Hz, 1H), 2.30–2.23 (m, 2H), 2.11–2.06 (m, 2H), 1.99–1.92 (m, 1H), 1.74–1.66 (m, 2H), 1.57–1.41 (m, 5H); ^{13}C NMR (100.6 MHz, $CDCl_3$): δ (ppm) = 138.6, 137.1, 136.7, 114.9, 62.9, 53,1, 49.8, 47.0, 45.6, 45.2, 37.9, 33.5, 28.9, 26.5, 26.2; HRMS (ESI, Q-ToF) *m/z*: calculated for $C_{15}H_{22}NaO_2S$ $[M+Na]^+$ 289.1233, found: 289.1234; IR (neat): υ_{max} = 3063, 2935, 1640, 1456, 1300, 1216, 1138, 912 cm^{-1}.

Dihexenyl sulfone 2d: $\boldsymbol{R_f}$ = 0.86 (20% EtOAc–petroleum ether).

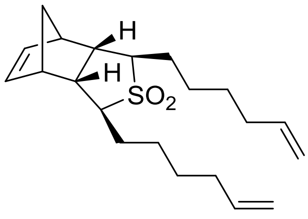

^{1}H NMR (500 MHz, $CDCl_3$): δ (ppm) = 6.23 (t, J = 1.8 Hz, 2H), 5.84–5.76 (m, 2H), 5.02 (dq, J = 17.1, 1.6 Hz, 2H), 4.98–4.95 (m, 2H), 3.00 (br s, 2H), 2.32–2.23 (m, 4H), 2.11–2.06 (m, 4H), 2.00–1.93 (m, 2H), 1.73–1.66 (m, 3H), 1.58–1.49 (m, 4H), 1.48–1.41 (m, 5H); ^{13}C NMR (125.7 MHz, $CDCl_3$): δ (ppm) = 138.6, 136.9, 114.9, 62.9, 49.9, 45.6, 45.6, 33.5, 28.9, 26.6, 26.3; HRMS (ESI, Q-ToF) *m/z*: calculated for $C_{21}H_{32}NaO_2S$ $[M+Na]^+$ 371.2015, found: 371.2014; IR (neat): υ_{max} = 3076, 2935, 1640, 1458, 1296, 1133, 911 cm^{-1}.

Synthesis of the tricyclic sulfone 10

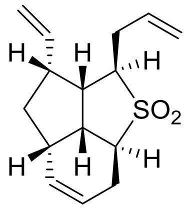

A solution of diallyl sulfone **2a** (150 mg, 0.57 mmol) in dry CH_2Cl_2 (40 mL) was degassed with nitrogen for 5 min and purged with ethylene for another 10 min. Next, Grubbs 1st generation catalyst (46.70 mg, 10 mol%) was added at rt in the presence of ethylene gas and the resulting reaction mixture was refluxed for 5 h. After completion of the reaction, the solvent was removed and the crude product was purified by silica gel column chromatography (12% EtOAc–petroleum ether) to get the tricyclic sulfone **10** (72 mg, 48%) as a white solid.

R*f* = 0.45 (20% EtOAc–petroleum ether); mp: 67–69 °C.

^{1}H NMR (500 MHz, $CDCl_3$): δ (ppm) = 5.89–5.72 (m, 4H), 5.21–5.07 (m, 4H), 3.16, 3.13 (ABq, J_{AB} = 7.1 Hz, 1H), 3.06–2.99 (m, 1H), 2.77–2.67 (m, 3H), 2.59–2.54 (m, 1H), 2.49–2.43 (m, 2H), 2.40–2.32 (m, 2H), 2.16–2.11 (m, 1H), 1.47, 1.42 (ABq, J_{AB} = 12.0 Hz, 1H); ^{13}C NMR (125.7 MHz, $CDCl_3$): δ (ppm) = 137.7 (d), 133.7 (d), 129.7 (d), 125.6 (d), 118.5 (t), 117.3 (t), 60.6 (d), 57.3 (d), 46.0 (d), 44.5 (d), 44.1 (d), 40.5 (d), 36.6 (t), 32.7 (t), 22.6 (t); HRMS (ESI, Q-ToF) *m/z*: calculated for $C_{15}H_{20}NaO_2S$ $[M+Na]^+$ 287.1076, found: 287.1075; IR (neat): υ_{max} = 3077, 2930, 1639, 1439, 1280, 1137, 918 cm^{-1}.

Synthesis of the tetracyclic sulfone 1b

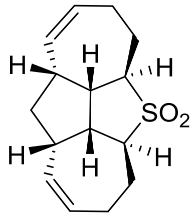

A solution of dibutenylated sulfone **2b** (25 mg, 0.08 mmol) in dry toluene (25 mL) was degassed with nitrogen for 10 min and purged with ethylene gas for another 10 min. Subsequently, G-II catalyst (7.26 mg, 10 mol%) was added in the presence of ethylene gas and the resulting reaction mixture was refluxed for 4 h. After completion of the reaction, the solvent was removed and the crude product was purified by silica gel column chromatography (15% EtOAc–petroleum ether) to obtain the desired tetracyclic sulfone **1b** (22 mg, 97%) as a white solid.

R*f* = 0.15 (10% EtOAc–petroleum ether); mp: 198–200 °C.

^{1}H NMR (500 MHz, $CDCl_3$): δ (ppm) = 5.62–5.56 (m, 2H), 5.42 (dt, *J* = 10.9, 2.0 Hz, 2H), 3.15–3.10 (m, 2H), 3.00–2.94 (m, 2H), 2.85–2.78 (m, 2H), 2.39–2.30 (m, 2H), 2.16–1.96 (m, 7H), 1.42, 1.36 (ABq, J_{AB} = 13.0 Hz, 1H); ^{13}C NMR (125.7 MHz, $CDCl_3$): δ (ppm) = 130.7 (d), 126.0 (d), 58.8 (d), 42.6 (d), 41.7 (d), 38.0 (t), 22.9 (t), 20.3 (t); HRMS (ESI, Q-ToF) *m/z*: calculated for $C_{15}H_{20}NaO_2S$ $[M+Na]^+$ 287.1076, found: 287.1078; IR (neat): υ_{max} = 3017, 2951, 2868, 1637, 1463, 1307, 1273, 1126 cm^{-1}.

Synthesis of the compounds 11, 1c and 12

A solution of dipentenylated sulfone **2c** (30 mg, 0.09 mmol) in dry toluene (30 mL) was degassed with nitrogen for 10 min and purged with ethylene gas for another 10 min. Next, Grubbs 2nd generation (G-II) catalyst (7.95 mg, 10 mol%) was added in the presence of ethylene gas and the resulting reaction mixture was refluxed for 9.5 h. After completion of the reaction, the solvent was evaporated and the crude product was purified by silica gel column chromatography (6% EtOAc-petroleum ether) to get the tricyclic sulfone **11** (18 mg, 60%) as a white solid. Continued elution of the column with 10% EtOAc–petroleum ether furnished the desired tetracyclic sulfone **1c** (8.70 mg, 32%) as a white solid. Also, a minor amount of the ring-opened product **12** (2 mg, 6%) was isolated as a white solid.

Tricyclic sulfone 11: $\boldsymbol{R_f}$ = 0.32 (10% EtOAc–petroleum ether); mp: 81–83 ^{o}C.

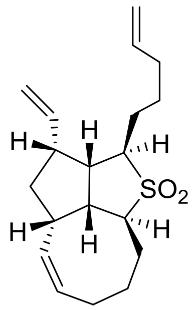

^{1}H NMR (500 MHz, $CDCl_3$): δ (ppm) = 5.81–5.72 (m, 2H), 5.68–5.62 (m, 1H), 5.36 (dd, J = 10.7, 3.1 Hz, 1H), 5.14–5.09 (m, 2H), 5.01 (dd, J = 17.1, 1.4 Hz, 1H), 4.96 (d, J = 10.1 Hz, 1H), 3.12–3.06 (m, 2H), 2.81–2.75 (m, 2H), 2.71–2.67 (m, 1H), 2.54–2.48 (m, 1H), 2.36 (d, J = 14.1 Hz, 1H), 2.18–2.12 (m, 1H), 2.08–1.95 (m, 5H), 1.92–1.86 (m, 1H), 1.81–1.73 (m, 2H), 1.64–1.50 (m, 3H), 1.42–1.33 (m, 1H); ^{13}C NMR (100.6 MHz, $CDCl_3$): δ (ppm) = 138.2 (d), 137.3 (d), 133.2 (d), 130.7 (d), 116.8 (t), 115.2 (t), 59.4 (d), 59.1 (d), 49.9 (d), 48.3 (d), 46.2 (d), 39.8 (d), 38.3 (t), 33.7 (t), 28.4 (t), 27.3 (t), 26.9 (t), 25.9 (t), 24.5 (t); HRMS (ESI, Q-ToF) *m/z*: calculated for $C_{19}H_{28}NaO_2S$ $[M+Na]^+$ 343.1702, found: 343.1703; IR (neat): υ_{max} = 3072, 2925, 1640, 1458, 1282, 1128, 909 cm^{-1}.

Tetracyclic sulfone 1c: $\boldsymbol{R_f}$ = 0.13 (10% EtOAc–petroleum ether); mp: 128–130 ^{o}C.

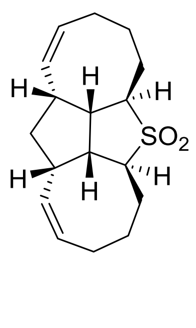

^{1}H NMR (500 MHz, $CDCl_3$): δ (ppm) = 5.71–5.65 (m, 2H), 5.42 (dd, J = 10.8, 4.8 Hz, 2H), 3.22 (br s, 2H), 3.03 (t, J = 8.7 Hz, 2H), 2.85–2.81 (m,

2H), 2.32–2.29 (m, 2H), 2.25–2.20 (m, 1H), 2.17–2.07 (m, 5H), 1.99–1.94 (m, 3H), 1.75–1.61 (m, 3H); ^{13}C NMR (125.7 MHz, $CDCl_3$): δ (ppm) = 133.3 (d), 131.0 (d), 59.3 (d), 50.6 (d), 40.0 (t), 39.9 (d), 27.6 (t), 27.1 (t), 24.5 (t); HRMS (ESI, Q-ToF) *m/z*: calculated for $C_{17}H_{24}NaO_2S$ $[M+Na]^+$ 315.1389, found: 315.1390; IR (neat): υ_{max} = 3015, 2933, 2863, 1454, 1439, 1292, 1128 cm^{-1}

Ring-opened compound 12: $\boldsymbol{R_f}$ = 0.37 (10% EtOAc–petroleum ether); mp: 126–128 °C.

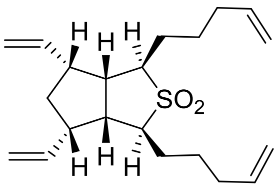

^{1}H NMR (500 MHz, $CDCl_3$): δ (ppm) = 5.82–5.74 (m, 4H), 5.17–5.13 (m, 4H), 5.01 (d, *J* = 17.1 Hz, 2H), 4.96 (d, *J* = 10.1 Hz, 2H), 2.82–2.79 (m, 4H), 2.45–2.40 (m, 2H), 2.07 (q, *J* = 6.9 Hz, 4H), 1.94–1.86 (m, 2H), 1.84–1.75 (m, 3H), 1.71–1.61 (m, 5H); ^{13}C NMR (125.7 MHz, $CDCl_3$): δ (ppm) = 138.2 (d), 136.4 (d), 117.1 (t), 115.2 (t), 60.1 (d), 46.2 (d), 45.8 (d), 34.1 (t), 33.6 (t), 27.6 (t), 25.9 (t); HRMS (ESI, Q-ToF) *m/z*: calculated for $C_{21}H_{32}NaO_2S$ $[M+Na]^+$ 371.2015, found: 371.2014; IR (neat): υ_{max} = 3074, 2922, 2858, 1640, 1457, 1281, 1126, 912 cm^{-1}.

Synthesis of the compound 13

A solution of the dihexenyl sulfone **2d** (21 mg, 0.06 mmol) in dry toluene (25 mL) was degassed with nitrogen for 15 min and purged with ethylene for another 5 min. Subsequently, G-II catalyst (5.11 mg, 10 mol%) was added at rt in the presence of ethylene gas then, the resulting reaction mixture was heated to reflux for 19 h. After completion of the reaction, the solvent was removed in vacuo and the crude product was purified by silica gel column chromatography (4% EtOAc–petroleum ether) to deliver the ring-opened compound **13** (19 mg, 88%) as a white solid. $\boldsymbol{R_f}$ = 0.66 (8% EtOAc–petroleum ether).

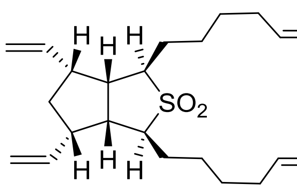

^{1}H NMR (500 MHz, $CDCl_3$): δ (ppm) = 5.83–5.75 (m, 4H), 5.17–5.14 (m, 4H), 4.99 (dd, *J* = 17.1, 1.7 Hz, 2H), 4.94 (d, *J* = 10.1 Hz, 2H), 2.82–2.77 (m, 4H), 2.43 (m, 2H), 2.18 (s, 2H),

2.04 (q, J = 6.8 Hz, 5H), 1.94–1.88 (m, 2H), 1.80–1.75 (m, 1H), 1.70–1.60 (m, 2H), 1.50–1.37 (m, 6H); ^{13}C NMR (100.6 MHz, $CDCl_3$): δ (ppm) = 138.6, 136.2, 116.9, 114.5, 59.9, 46.0, 45.6, 33.9, 33. 4, 28.7, 27.9, 26.1; HRMS (ESI, Q-ToF) *m/z*: calculated for $C_{23}H_{36}NaO_2S$ $[M+Na]^+$ 399.2328, found: 399.2327; IR (neat): υ_{max} = 2925, 1457, 1296, 1132 cm^{-1}.

References

1. Zhang, M. J.; Flynn, D. L.; Hanson, P. R. *J. Org. Chem.* **2007**, *72*, 3194–3198.

doi: 10.1021/Jo0620260

2. Bloch, R.; Abecassis, J. *Tetrahedron Lett.* **1982**, *23*, 3277–3280.

doi: 10.1016/S0040-4039(00)87591-7

3. Aitken, R. A.; Cadogan, J. I. G.; Gosney, I.; Newlands, S. F. *J. Chem. Soc., Perkin Trans. 1* **1994**, 2301–2308. doi: 10.1039/P19940002301

4. Bloch, R.; Abecassis, J.; Hassan, D. *J. Org. Chem.* **1985**, *50*, 1544–1545.

doi: 10.1021/Jo00209a040

5. Bloch, R.; Abecassis, J.; Hassan, D. *Can. J. Chem.* **1984**, *62*, 2019–2024.

doi: 10.1139/V84-345

6. Kotha, S.; Gunta, R. *Acta Cryst.* **2014**, *E70*, o1163–o1164.

doi: 10.1107/S1600536814022053

^{1}H, ^{13}C NMR and HRMS spectra of the compounds 2a–d, 8b–d, 1b, 1c and 10–13;

DDEPT-135 spectra of the selected compounds

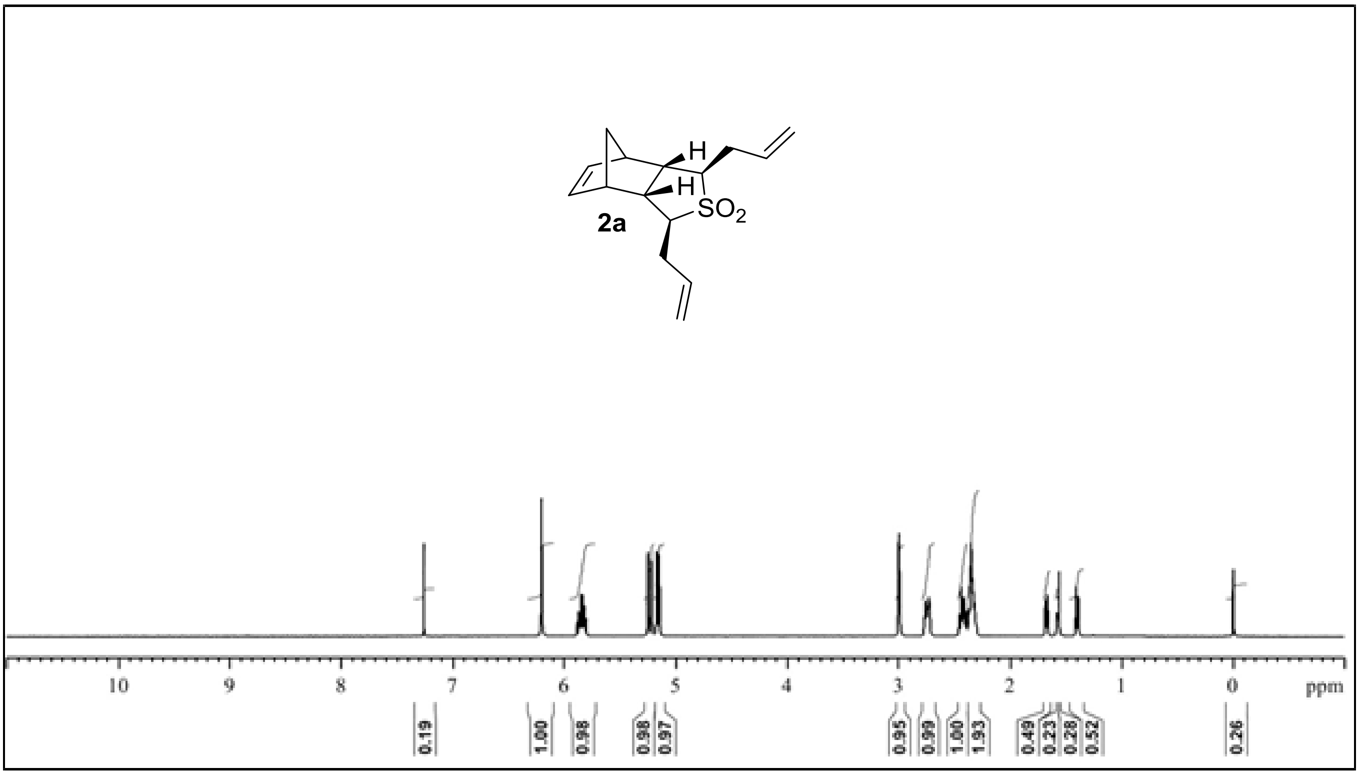

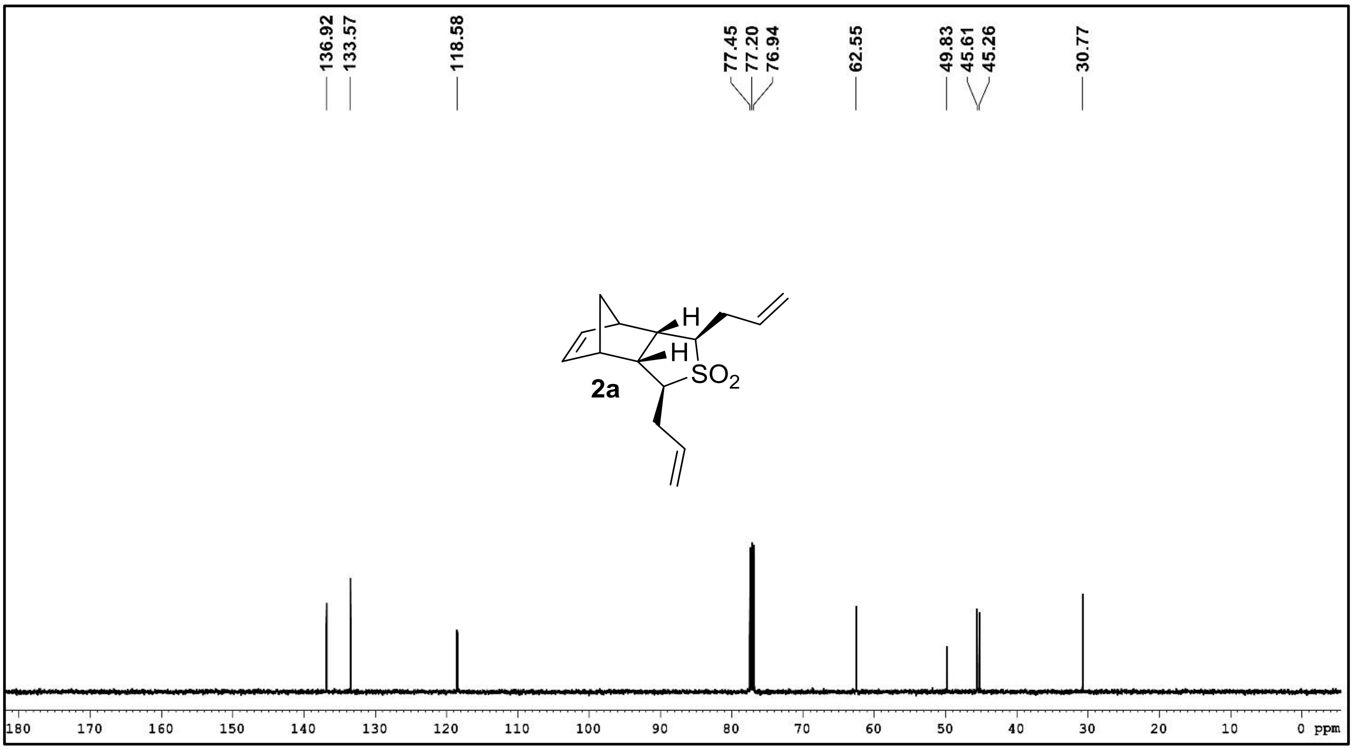

Acquisition Parameter

Source Type	ESI	Ion Polarity	Positive	Set Nebulizer	0.3 Bar
Focus	Active	Set Capillary	3500 V	Set Dry Heater	180 °C
Scan Begin	50 m/z	Set End Plate Offset	-500 V	Set Dry Gas	4.0 l/min
Scan End	700 m/z	Set Collision Cell RF	160.0 Vpp	Set Divert Valve	Source

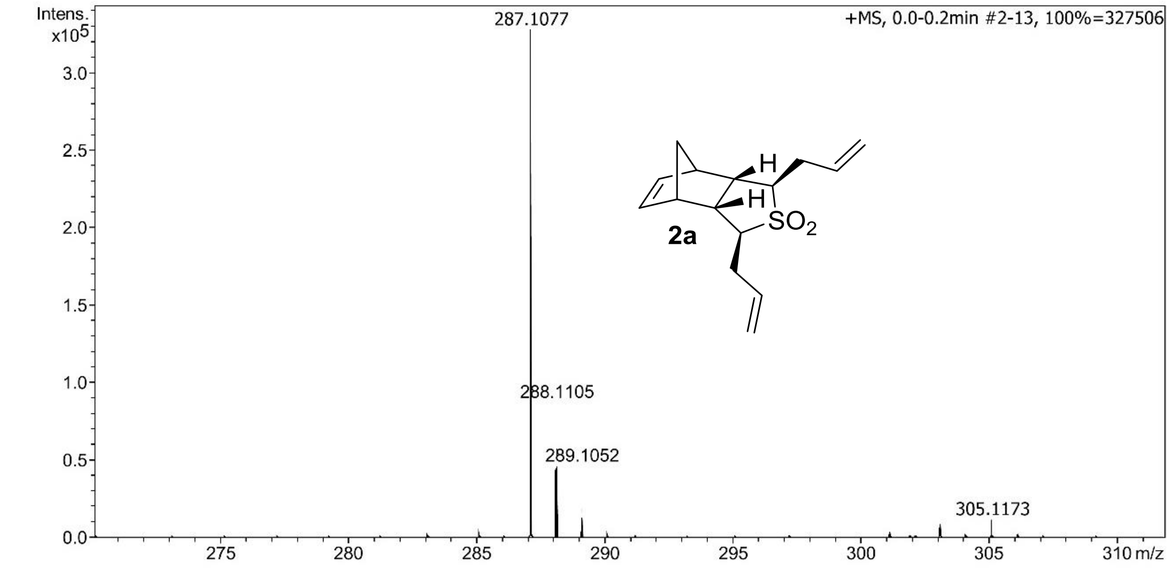

Meas. m/z	#	Ion Formula	m/z	err [ppm]	mSigma	# Sigma	Score	rdb	e⁻ Conf	N-Rule
287.1077	1	C15H20NaO2S	287.1076	0.1	21.4	1	100.00	5.5	even	ok

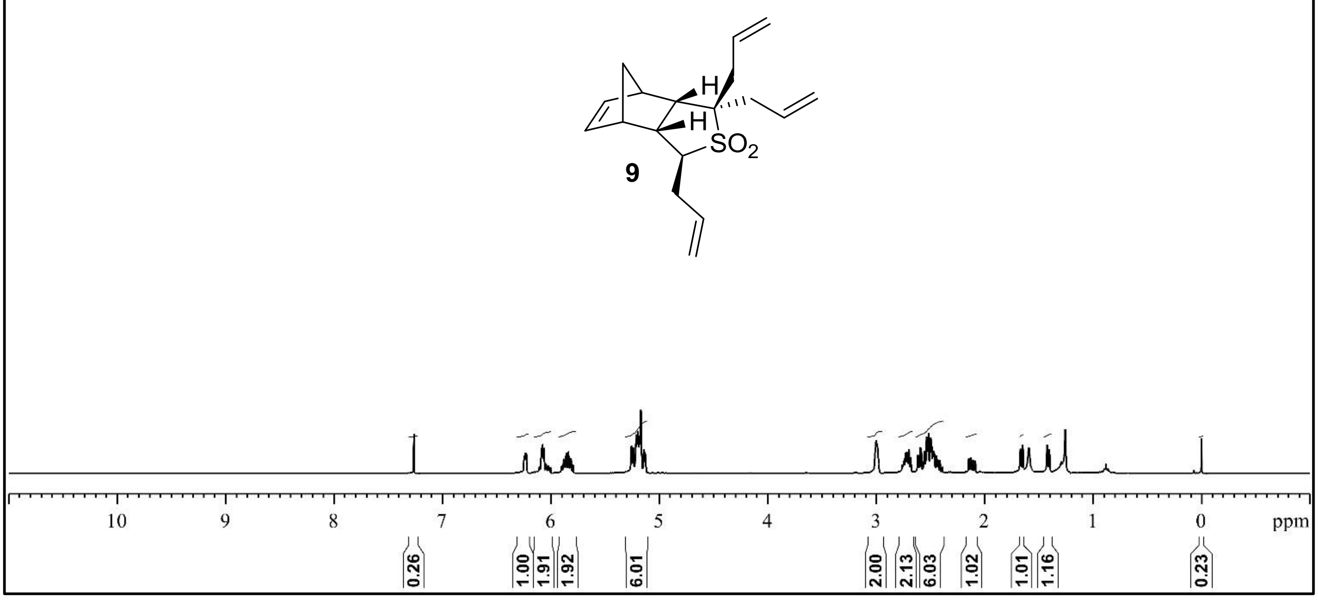

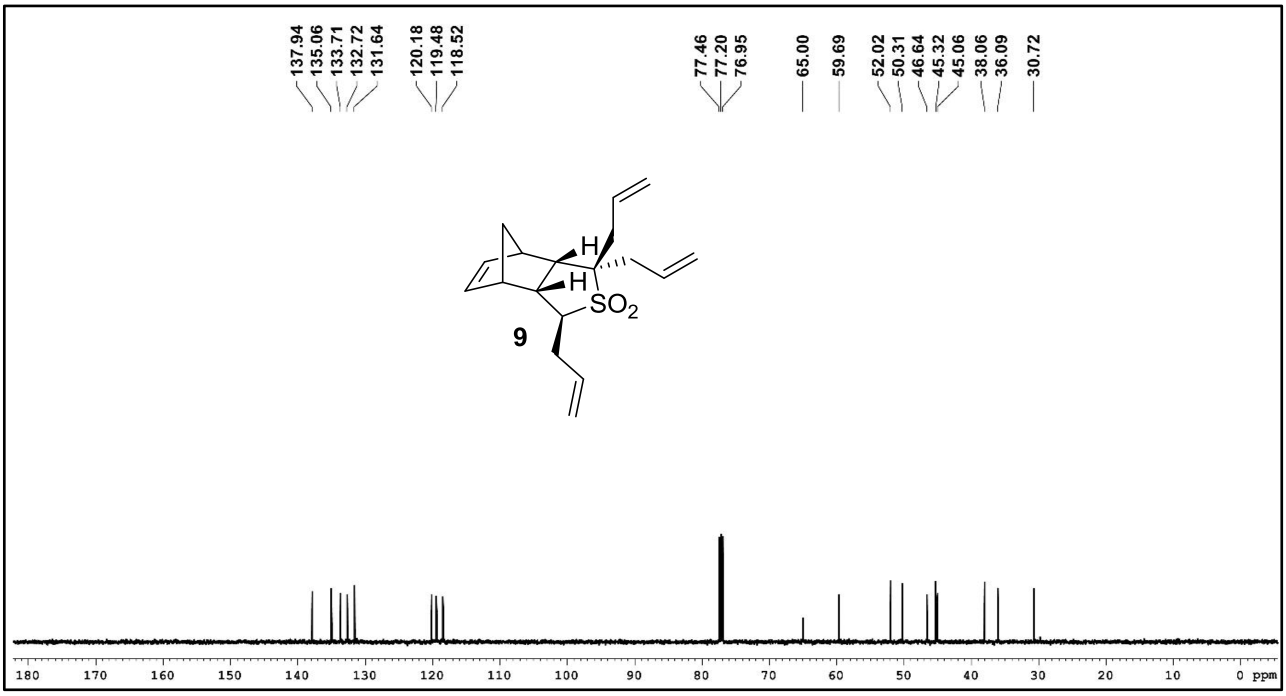

Acquisition Parameter

Source Type	ESI	Ion Polarity	Positive	Set Nebulizer	0.3 Bar
Focus	Active	Set Capillary	3500 V	Set Dry Heater	180 °C
Scan Begin	50 m/z	Set End Plate Offset	-500 V	Set Dry Gas	4.0 l/min
Scan End	500 m/z	Set Collision Cell RF	160.0 Vpp	Set Divert Valve	Source

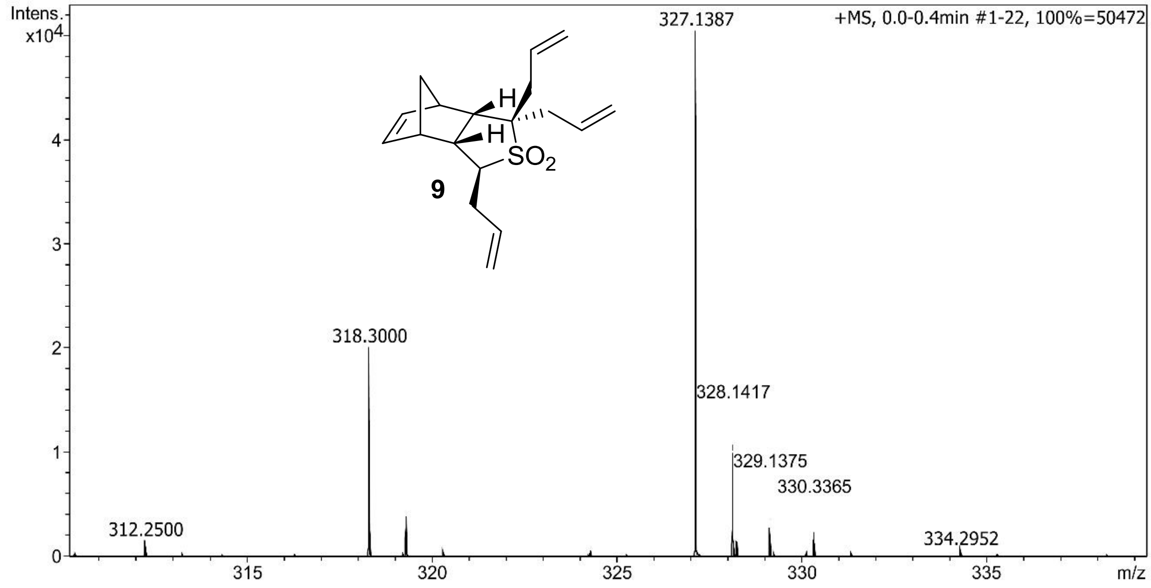

Meas. m/z	#	Ion Formula	m/z	err [ppm]	mSigma	# Sigma	Score	rdb	e⁻ Conf	N-Rule
327.1387	1	C18H24NaO2S	327.1389	-0.7	8.9	1	100.00	6.5	even	ok

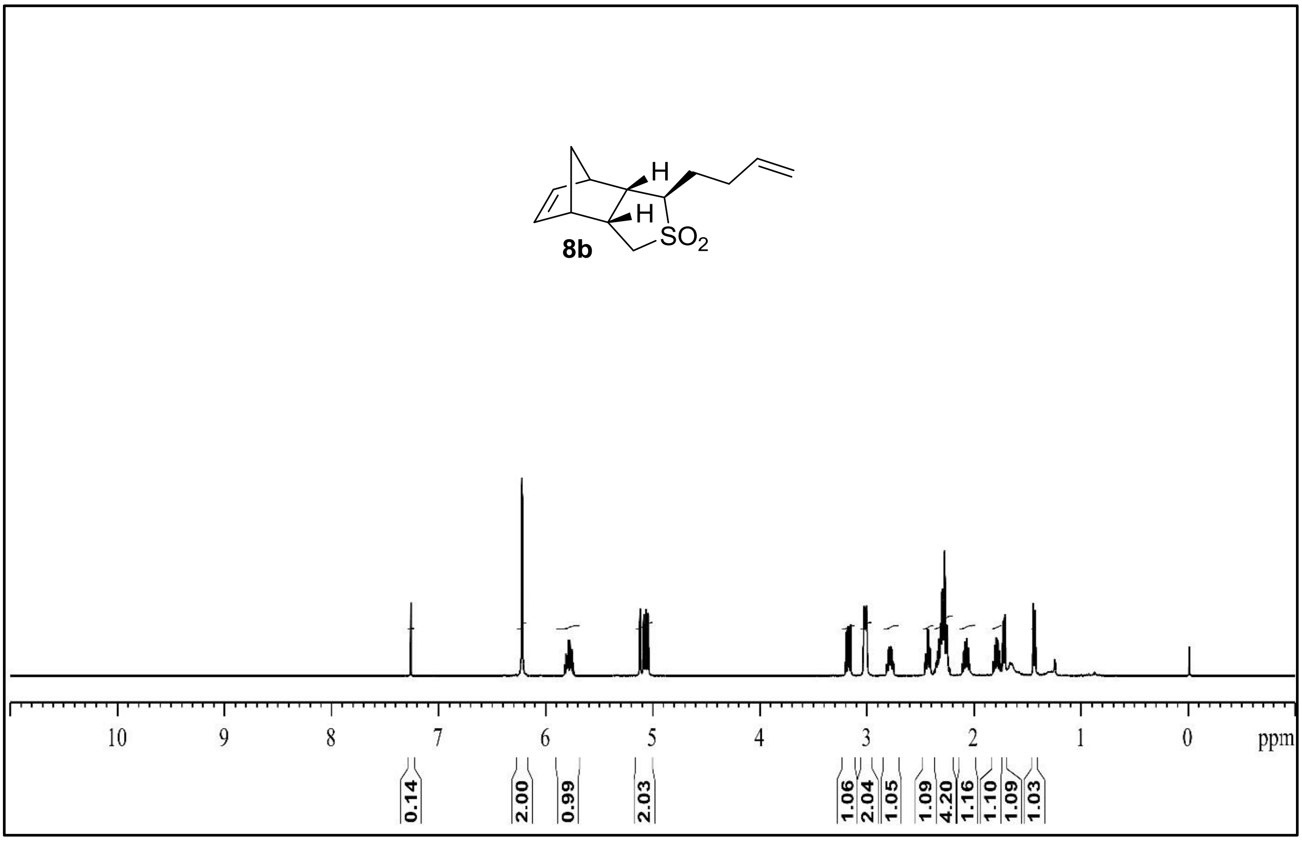
8b
SO2
10 9 8 7 6 5 4 3 2 1 0 ppm
0.14
2.00
0.99
2.03
1.06
2.04
1.05
1.09
4.20
1.16
1.10
1.09
1.03

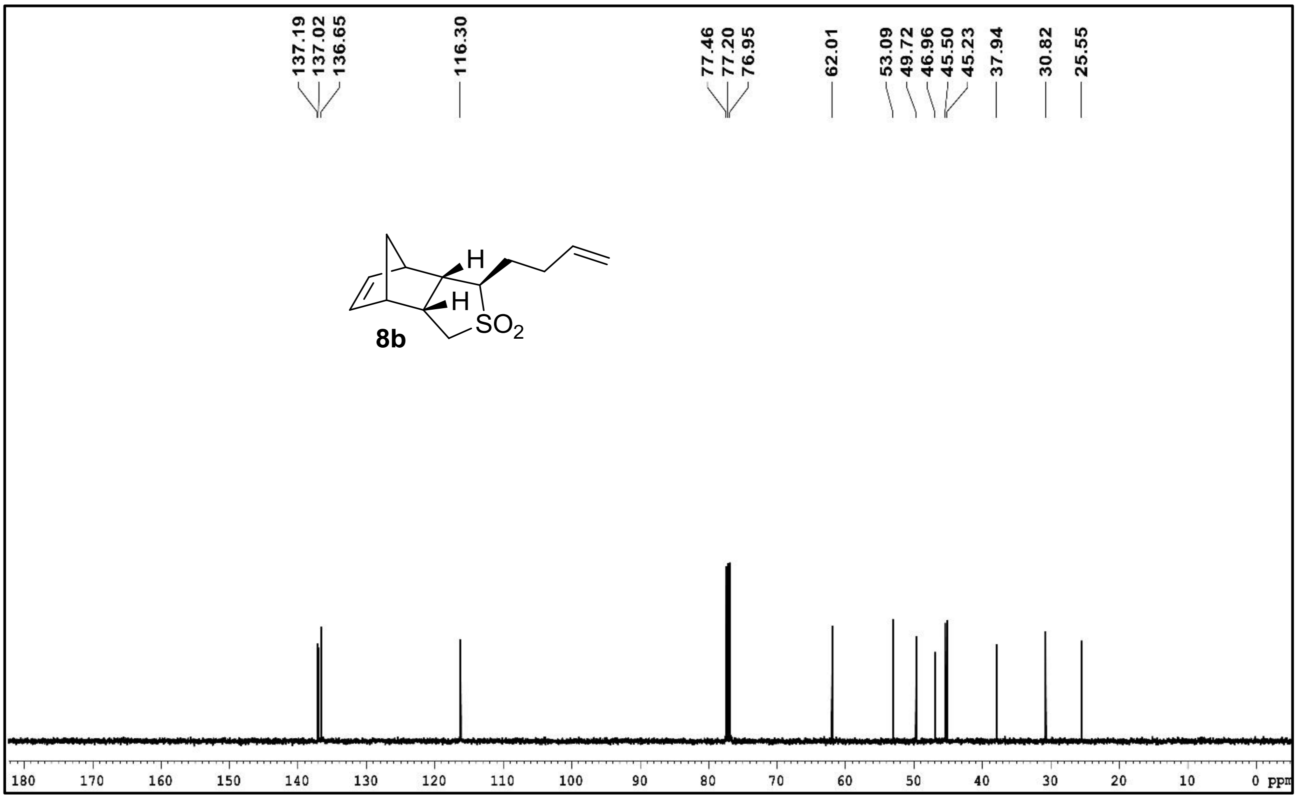
137.19
137.02
136.65
116.30
77.46
77.20
76.95
62.01
53.09
49.72
46.96
45.50
45.23
37.94
30.82
25.55
8b
SO2
180 170 160 150 140 130 120 110 100 90 80 70 60 50 40 30 20 10 0 ppm

Acquisition Parameter

Source Type	ESI	Ion Polarity	Positive	Set Nebulizer	0.3 Bar
Focus	Active	Set Capillary	3700 V	Set Dry Heater	180 °C
Scan Begin	50 m/z	Set End Plate Offset	-500 V	Set Dry Gas	4.0 l/min
Scan End	500 m/z	Set Collision Cell RF	1500.0 Vpp	Set Divert Valve	Source

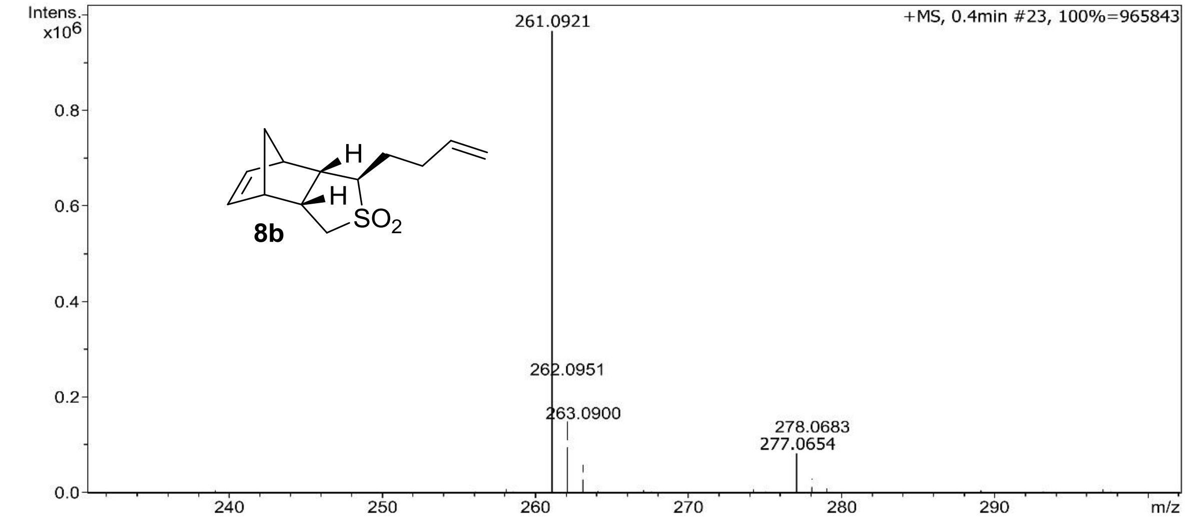

Meas. m/z	#	Ion Formula	m/z	err [ppm]	mSigma	# Sigma	Score	rdb	e⁻ Conf	N-Rule
261.0921	1	C13H18NaO2S	261.0920	-0.6	31.0	1	100.00	4.5	even	ok

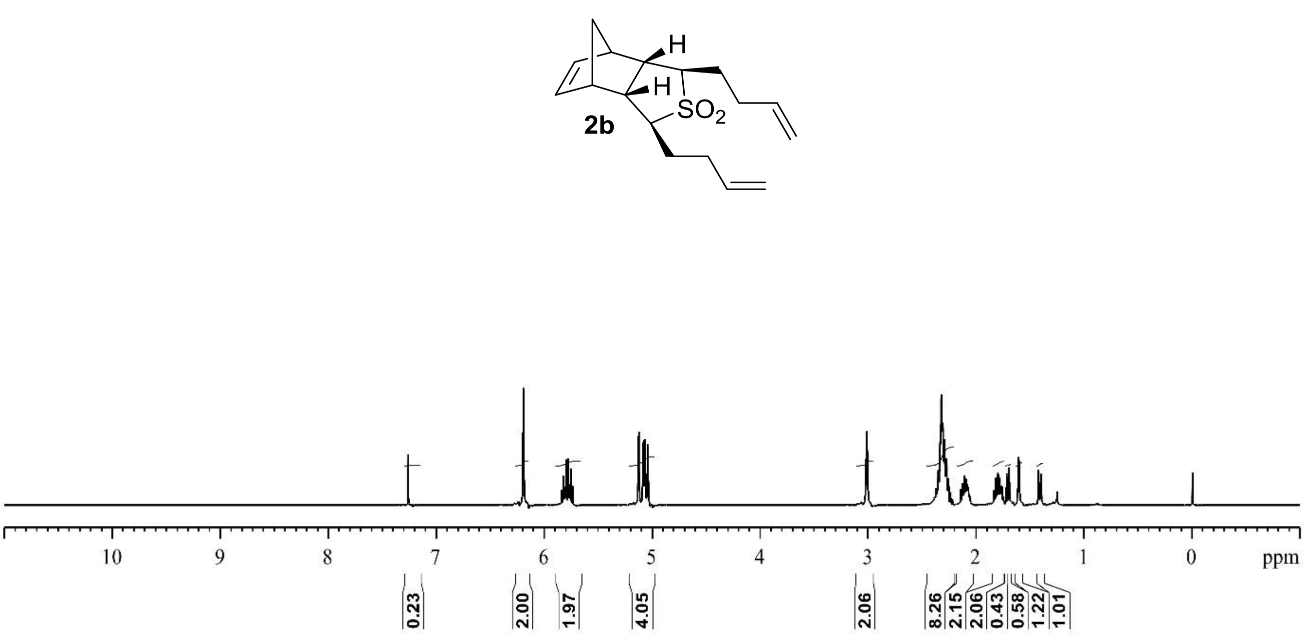

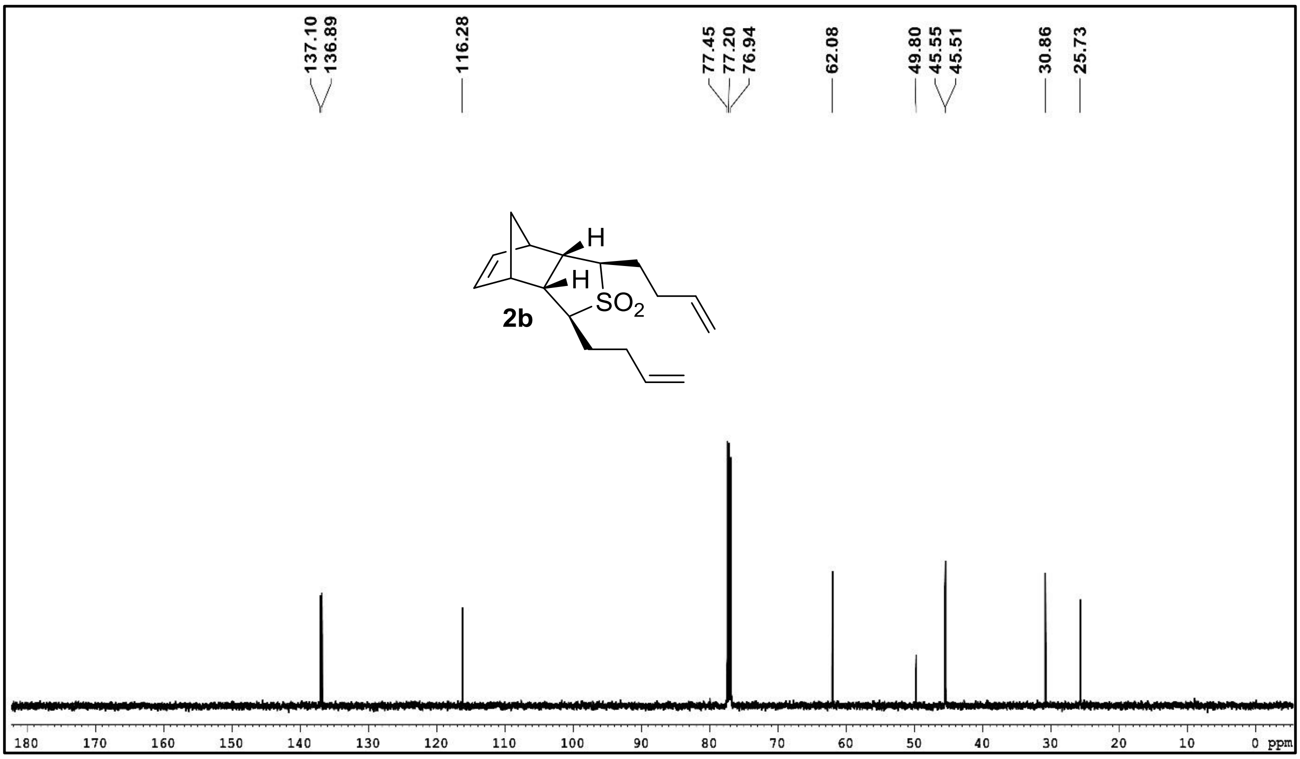

Acquisition Parameter

Source Type	ESI	Ion Polarity	Positive	Set Nebulizer	0.3 Bar
Focus	Active	Set Capillary	3700 V	Set Dry Heater	180 °C
Scan Begin	50 m/z	Set End Plate Offset	-500 V	Set Dry Gas	4.0 l/min
Scan End	500 m/z	Set Collision Cell RF	1500.0 Vpp	Set Divert Valve	Source

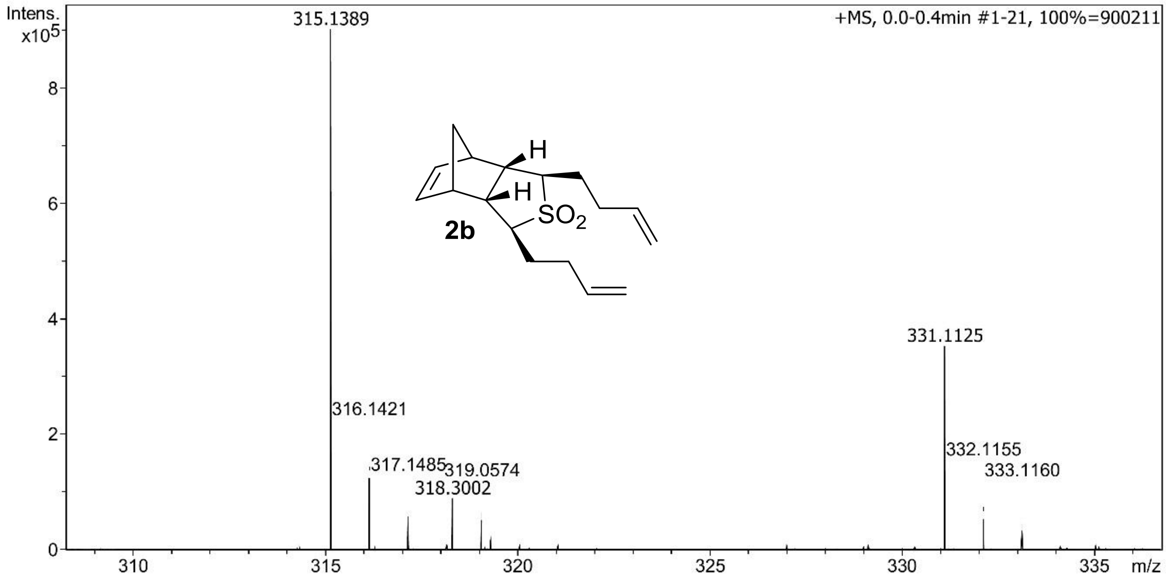

Meas. m/z	#	Ion Formula	m/z	err [ppm]	mSigma	# Sigma	Score	rdb	e⁻ Conf	N-Rule
315.1389	1	C17H24NaO2S	315.1389	-0.1	44.8	1	100.00	5.5	even	ok

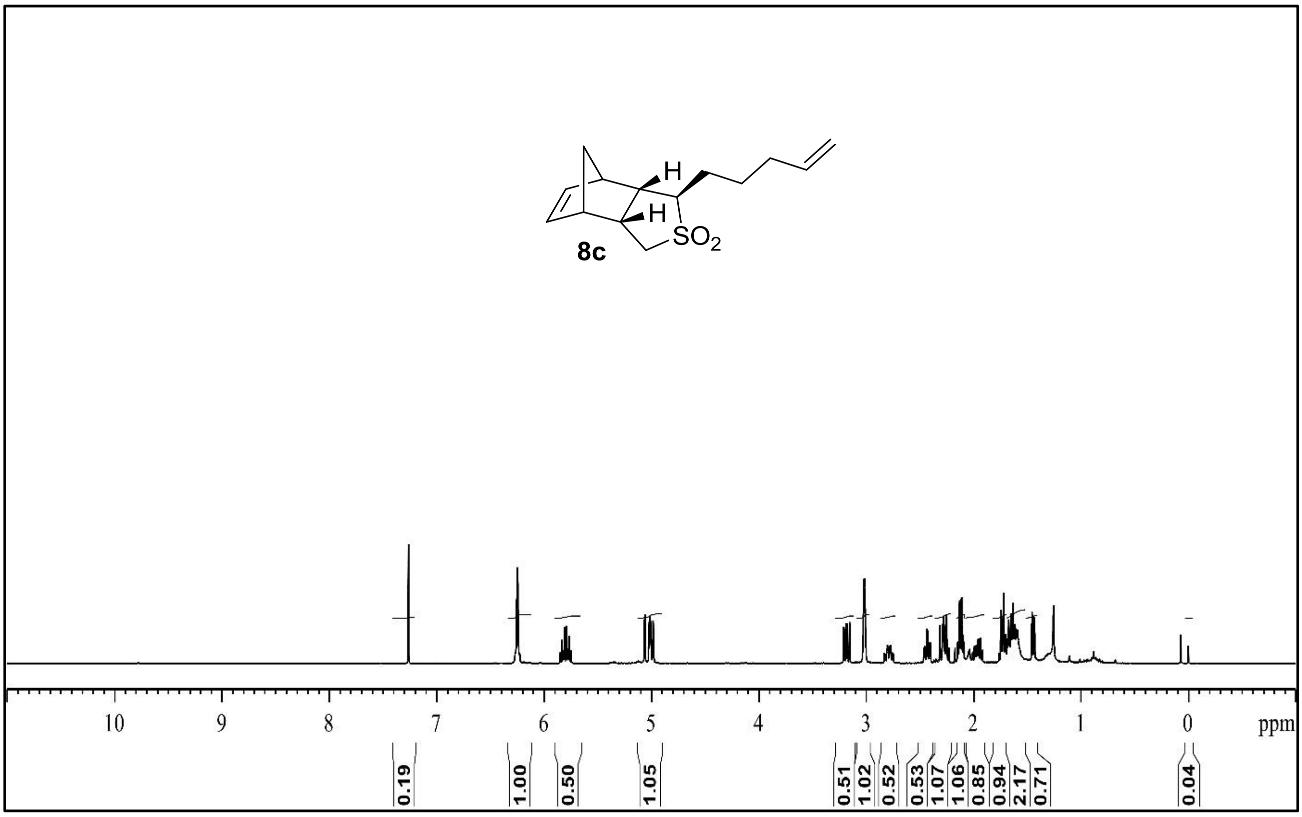
8c
SO2
ppm
10
9
8
7
6
5
4
3
2
1
0
0.19
1.00
0.50
1.05
0.51
1.02
0.52
0.53
1.07
1.06
0.85
0.94
2.17
0.71
0.04

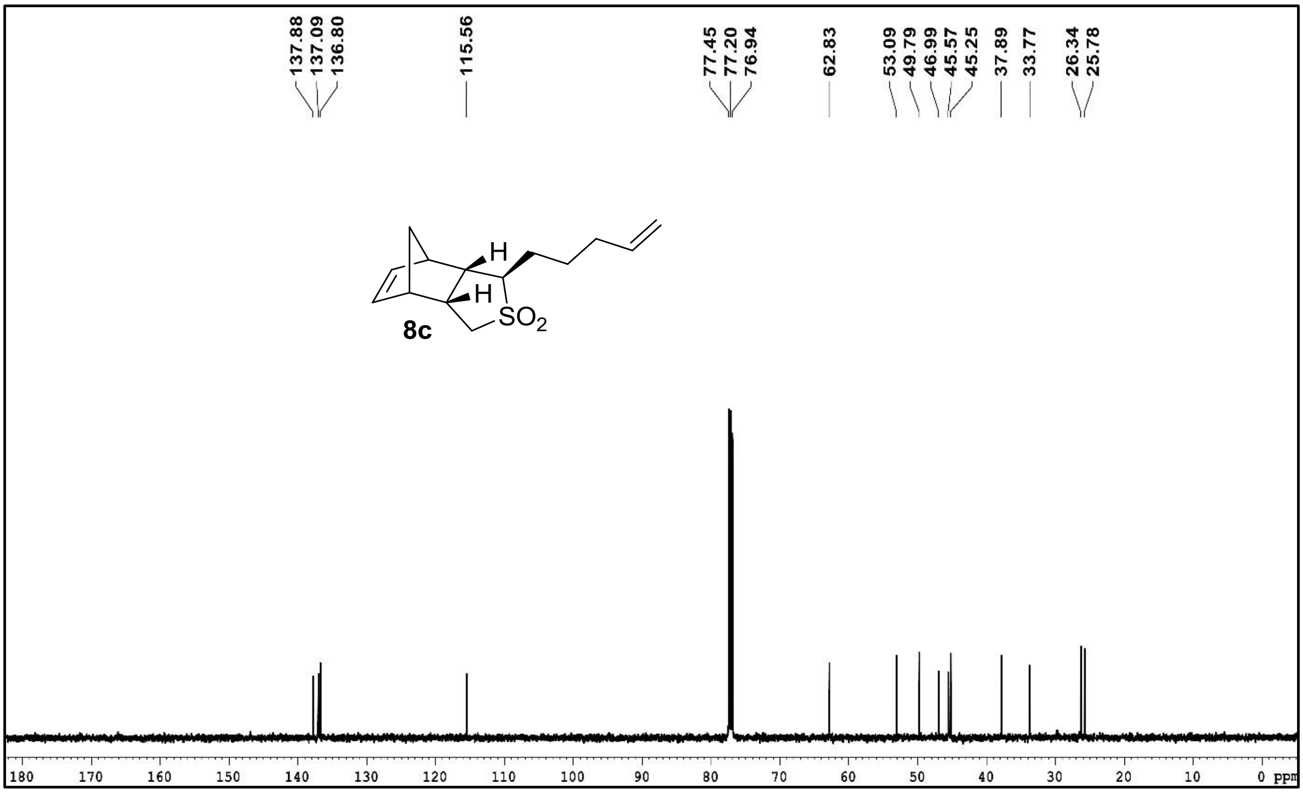
137.88
137.09
136.80
115.56
77.45
77.20
76.94
62.83
53.09
49.79
46.99
45.57
45.25
37.89
33.77
26.34
25.78
8c
SO2
180
170
160
150
140
130
120
110
100
90
80
70
60
50
40
30
20
10
0 ppm

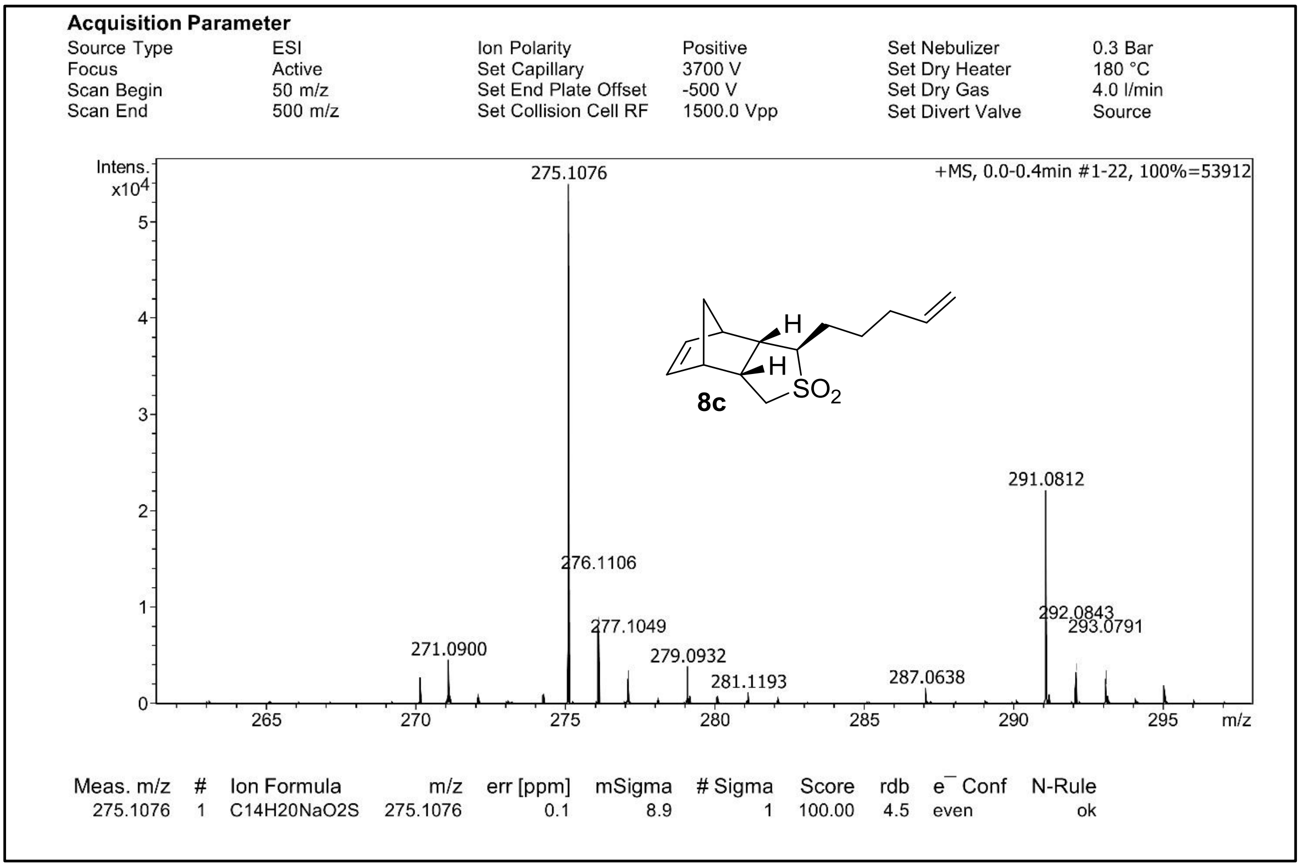
Acquisition Parameter
Source Type ESI
Focus Active
Scan Begin 50 m/z
Scan End 500 m/z
Ion Polarity Positive
Set Capillary 3700 V
Set End Plate Offset -500 V
Set Collision Cell RF 1500.0 Vpp
Set Nebulizer 0.3 Bar
Set Dry Heater 180 °C
Set Dry Gas 4.0 l/min
Set Divert Valve Source
Intens. x10^4
+MS, 0.0-0.4min #1-22, 100%=53912
275.1076
276.1106
277.1049
279.0932
281.1193
271.0900
287.0638
291.0812
292.0843
293.0791
8c
SO2
H
H
m/z
Meas. m/z # Ion Formula m/z err [ppm] mSigma # Sigma Score rdb e⁻ Conf N-Rule
275.1076 1 C14H20NaO2S 275.1076 0.1 8.9 1 100.00 4.5 even ok

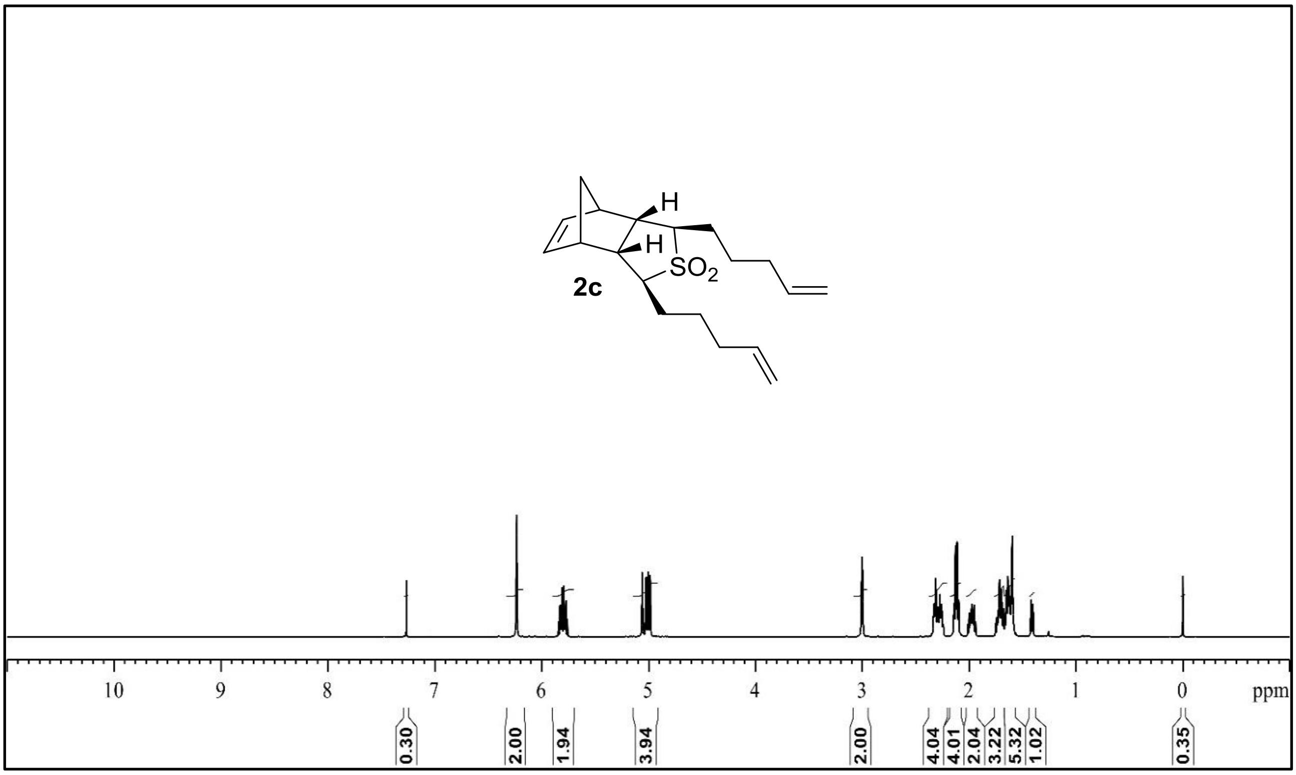
2c
SO2
H
H
ppm
0.30
2.00
1.94
3.94
2.00
4.04
4.01
2.04
3.22
5.32
1.02
0.35

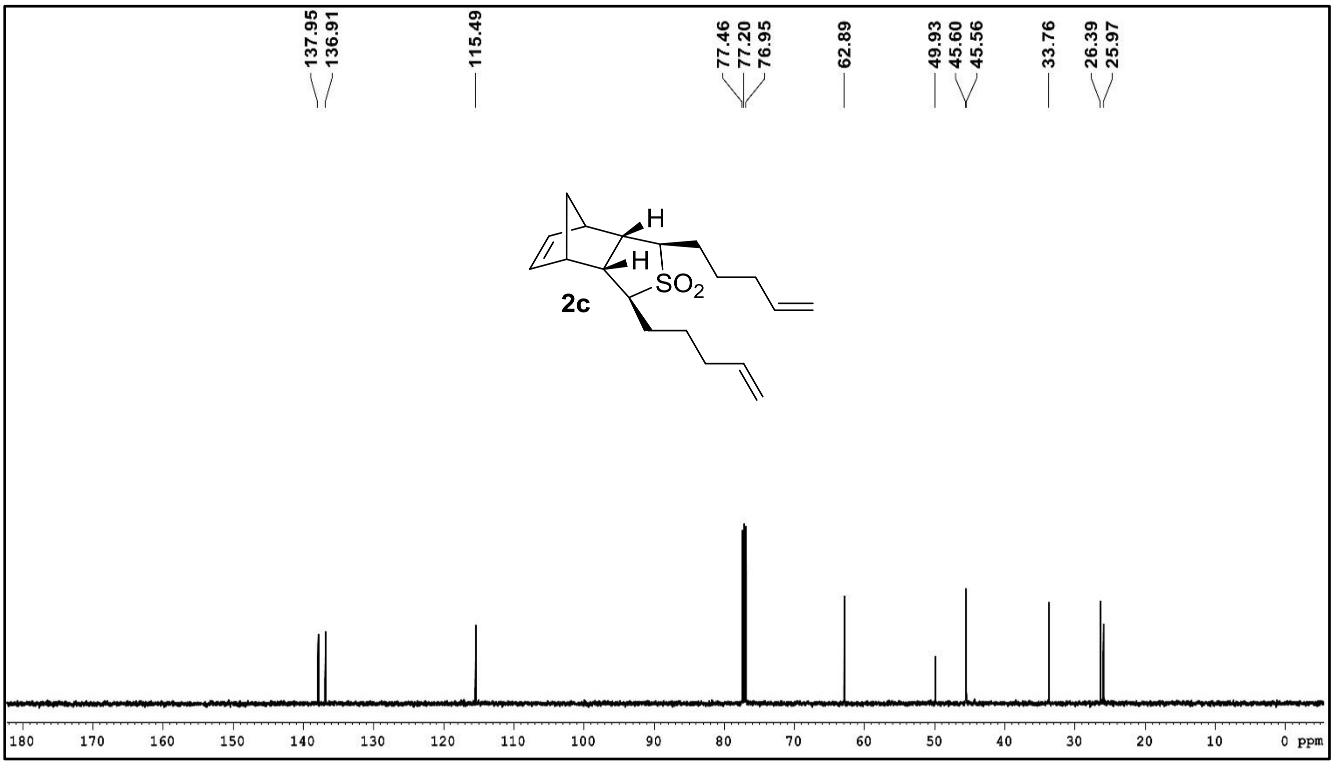

Acquisition Parameter

Source Type	ESI	Ion Polarity	Positive	Set Nebulizer	0.3 Bar
Focus	Active	Set Capillary	3700 V	Set Dry Heater	180 °C
Scan Begin	50 m/z	Set End Plate Offset	-500 V	Set Dry Gas	4.0 l/min
Scan End	500 m/z	Set Collision Cell RF	1500.0 Vpp	Set Divert Valve	Source

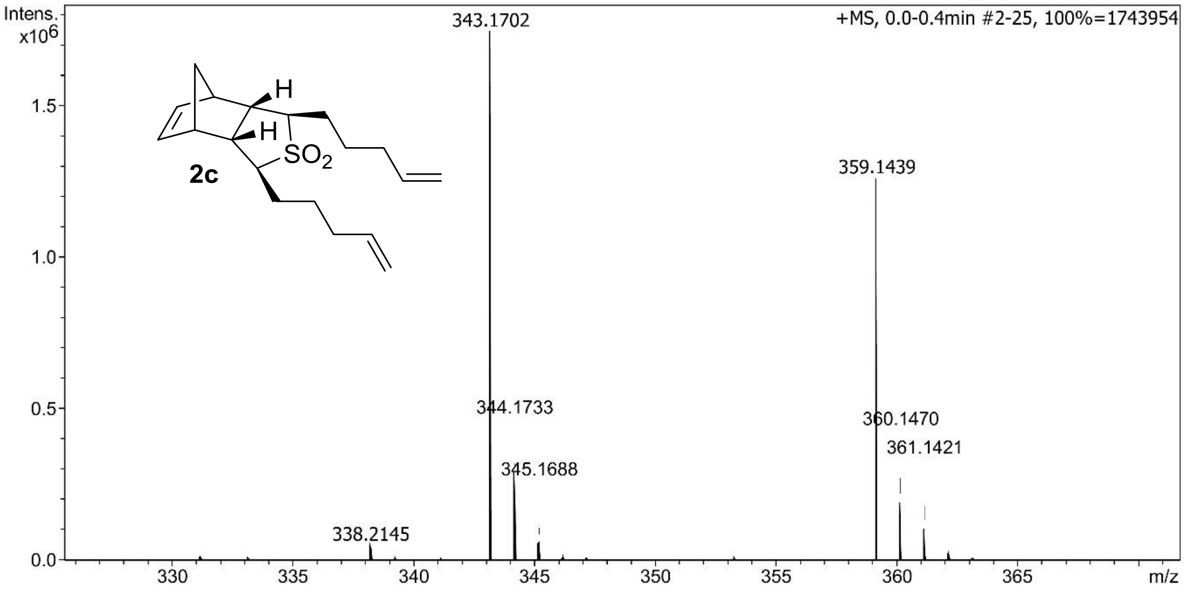

Meas. m/z	#	Ion Formula	m/z	err [ppm]	mSigma	# Sigma	Score	rdb	e⁻ Conf	N-Rule
343.1702	1	C19H28NaO2S	343.1702	-0.0	32.4	1	100.00	5.5	even	ok

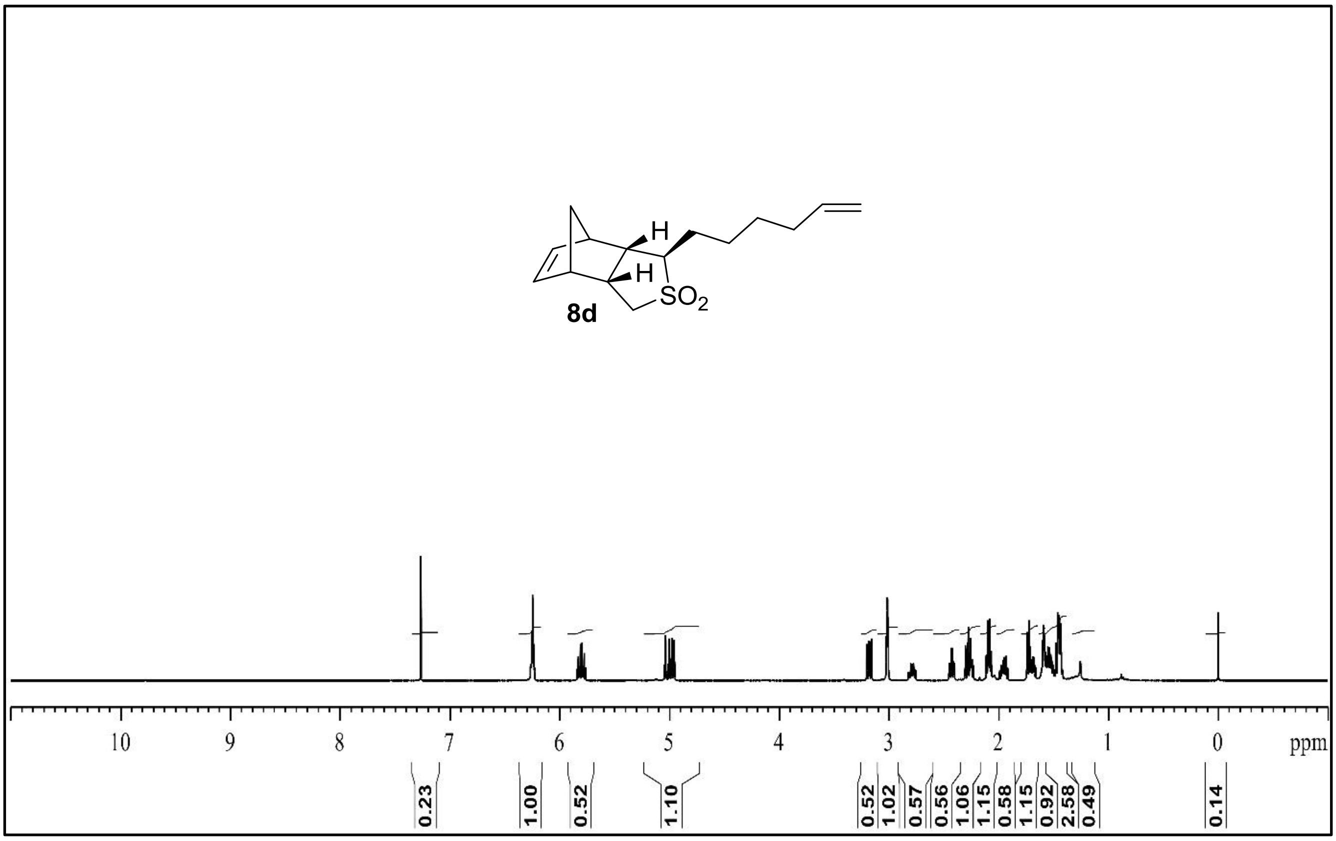
H
H
SO2
8d
10 9 8 7 6 5 4 3 2 1 0 ppm
0.23
1.00
0.52
1.10
0.52
1.02
0.57
0.56
1.06
1.15
0.58
1.15
0.92
2.58
0.49
0.14

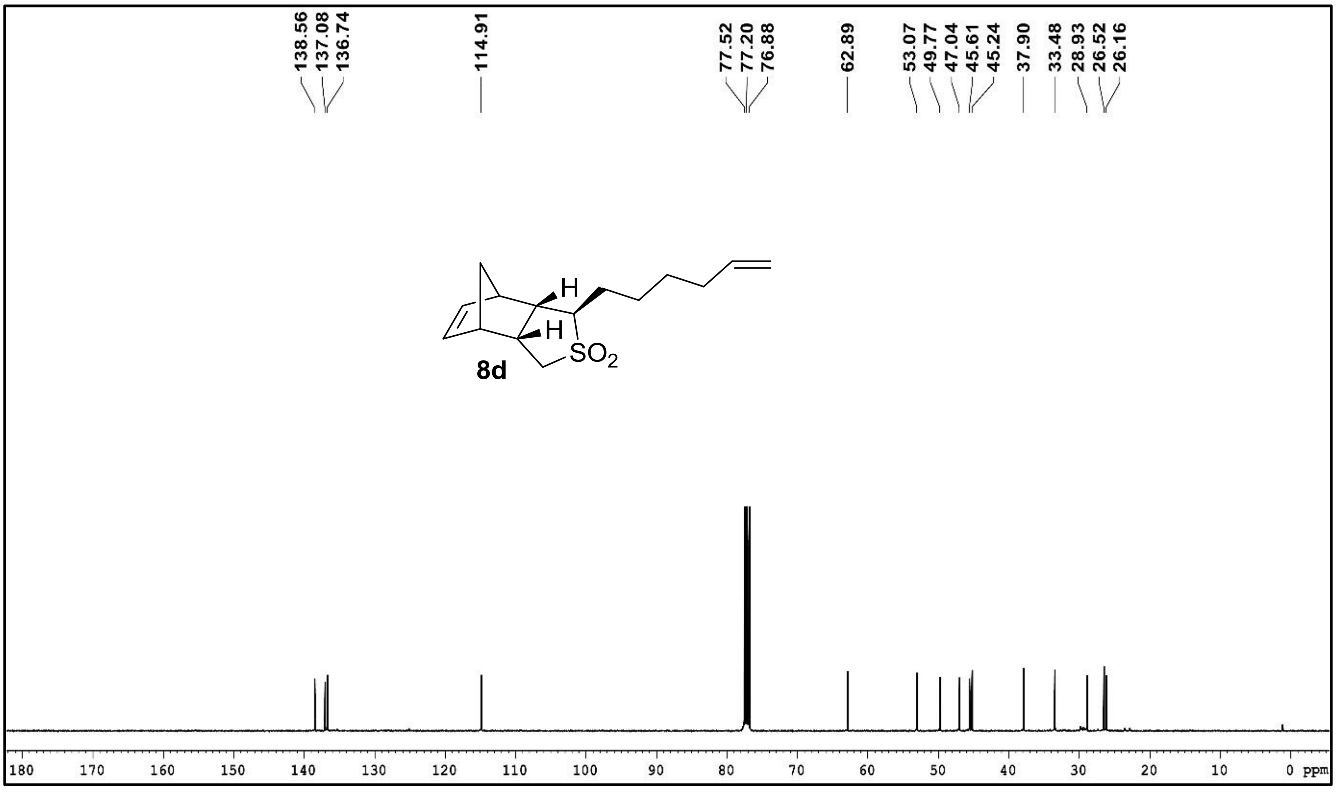
138.56
137.08
136.74
114.91
77.52
77.20
76.88
62.89
53.07
49.77
47.04
45.61
45.24
37.90
33.48
28.93
26.52
26.16
H
H
SO2
8d
180 170 160 150 140 130 120 110 100 90 80 70 60 50 40 30 20 10 0 ppm

Acquisition Parameter

Source Type	ESI	Ion Polarity	Positive	Set Nebulizer	0.3 Bar
Focus	Active	Set Capillary	3700 V	Set Dry Heater	180 °C
Scan Begin	50 m/z	Set End Plate Offset	-500 V	Set Dry Gas	4.0 l/min
Scan End	500 m/z	Set Collision Cell RF	1500.0 Vpp	Set Divert Valve	Source

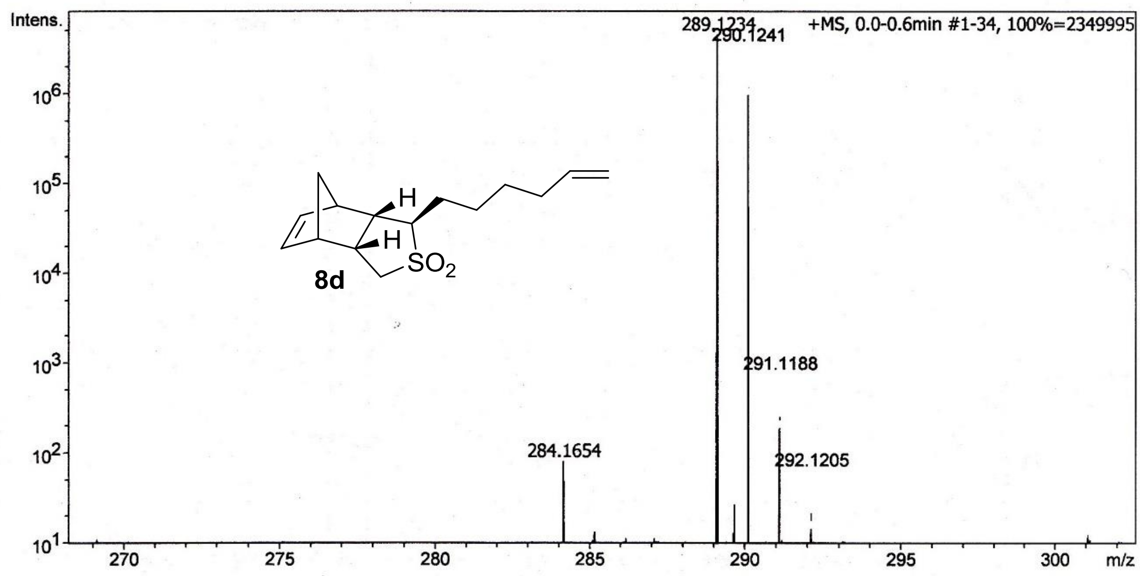

Meas. m/z	#	Ion Formula	m/z	err [ppm]	mSigma	# Sigma	Score	rdb	e⁻ Conf	N-Rule
289.1234	1	C15H22NaO2S	289.1233	-0.4	363.3	1	100.00	4.5	even	ok

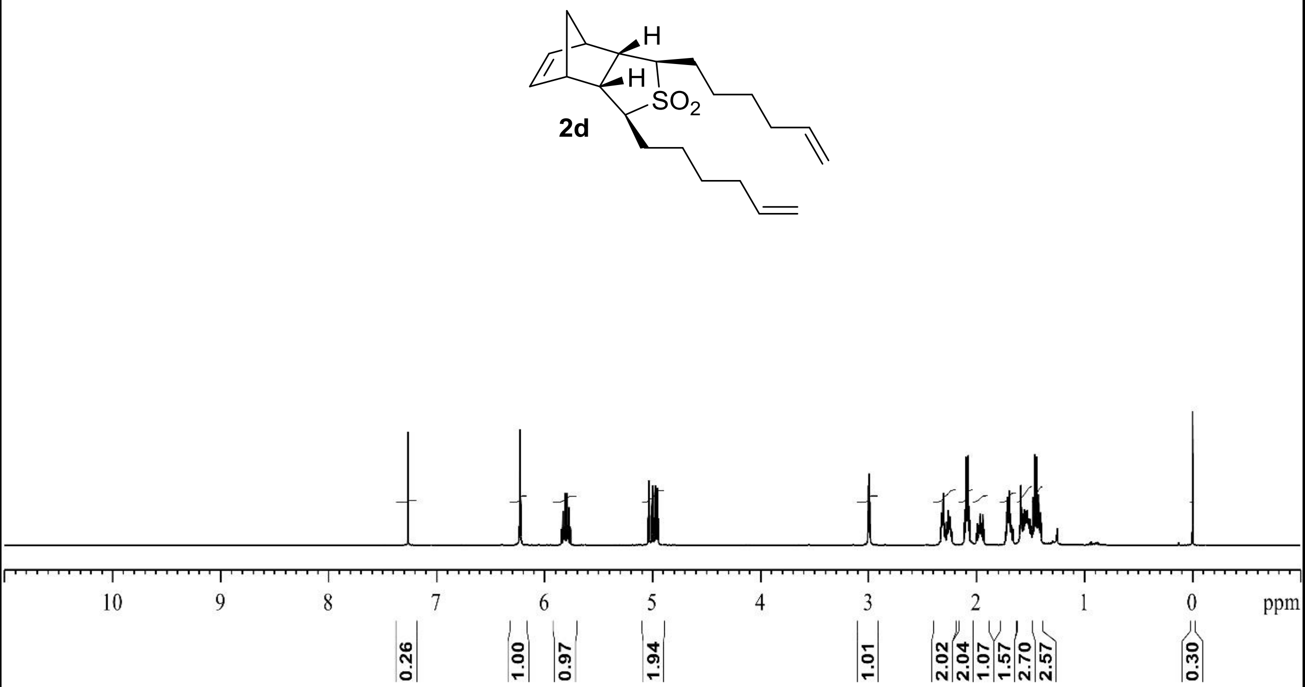

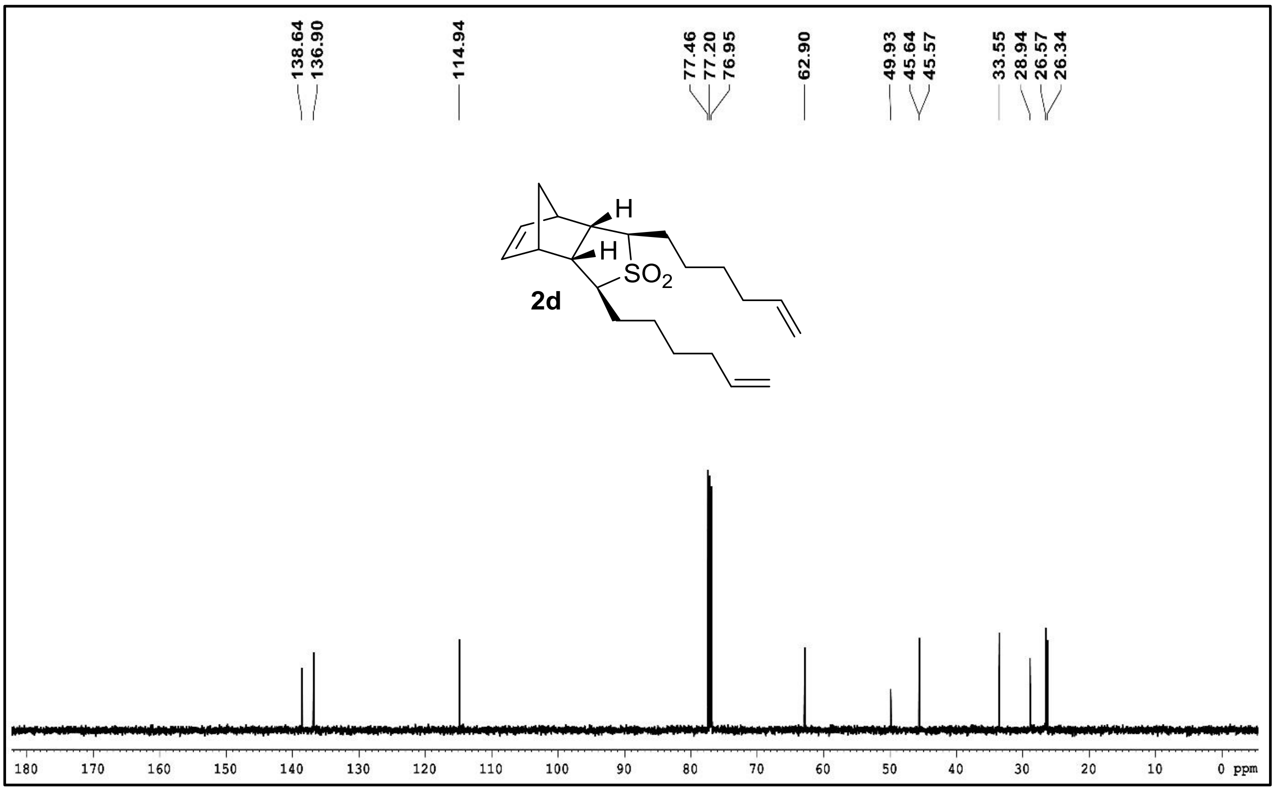

Acquisition Parameter

Source Type	ESI	Ion Polarity	Positive	Set Nebulizer	0.3 Bar
Focus	Active	Set Capillary	3700 V	Set Dry Heater	180 °C
Scan Begin	50 m/z	Set End Plate Offset	-500 V	Set Dry Gas	4.0 l/min
Scan End	500 m/z	Set Collision Cell RF	1500.0 Vpp	Set Divert Valve	Source

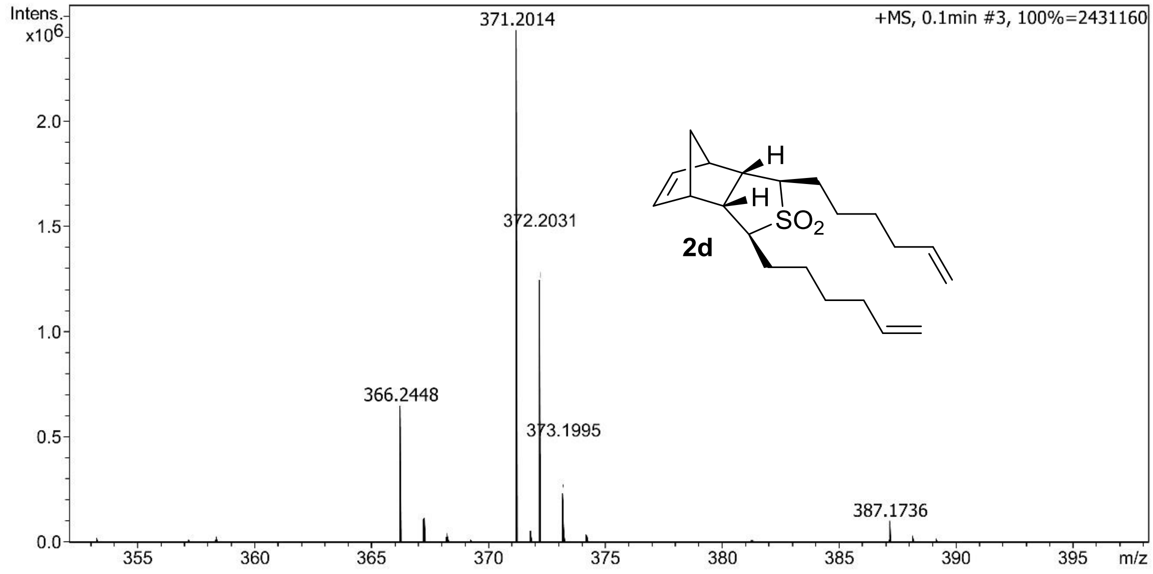

Meas. m/z	#	Ion Formula	m/z	err [ppm]	mSigma	# Sigma	Score	rdb	e⁻ Conf	N-Rule
371.2014	1	C21H32NaO2S	371.2015	-0.4	136.5	1	100.00	5.5	even	ok

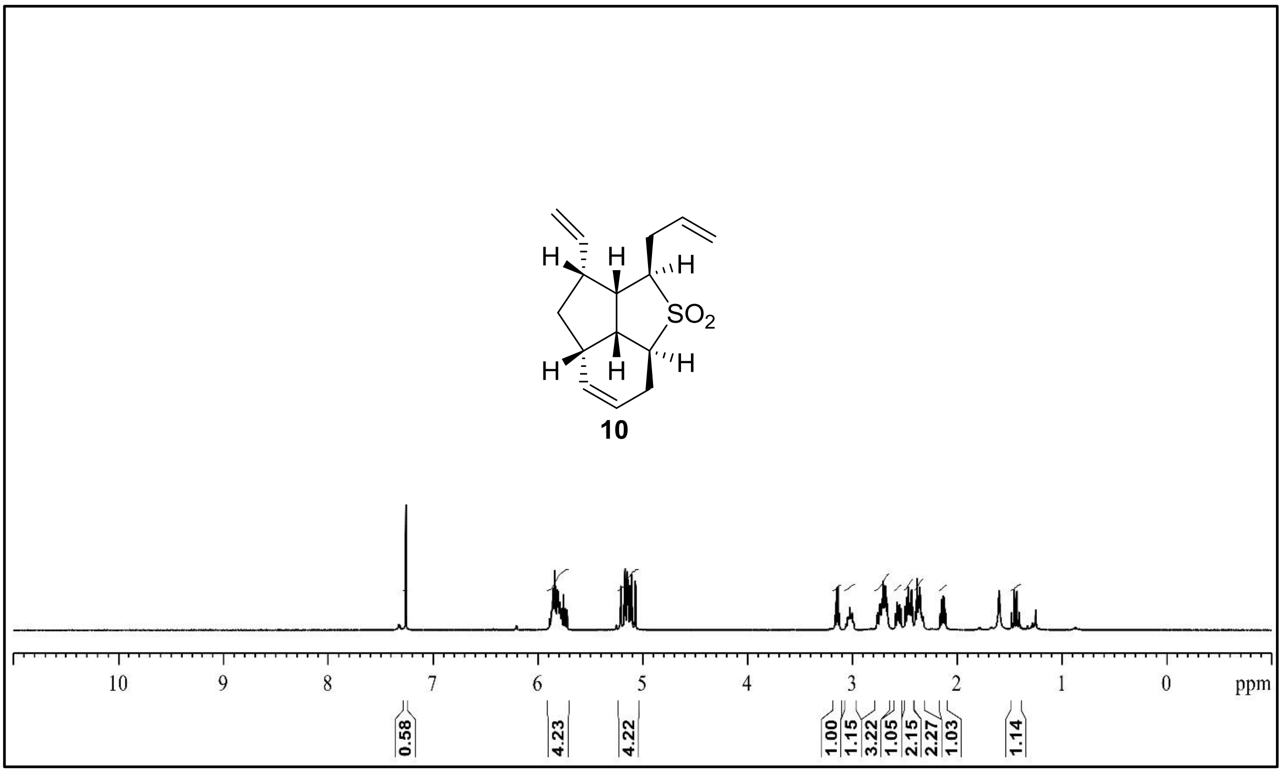

H
H
H
SO2
H
H
H
10
10
9
8
7
6
5
4
3
2
1
0
ppm
0.58
4.23
4.22
1.00
1.15
3.22
1.05
2.15
2.27
1.03
1.14

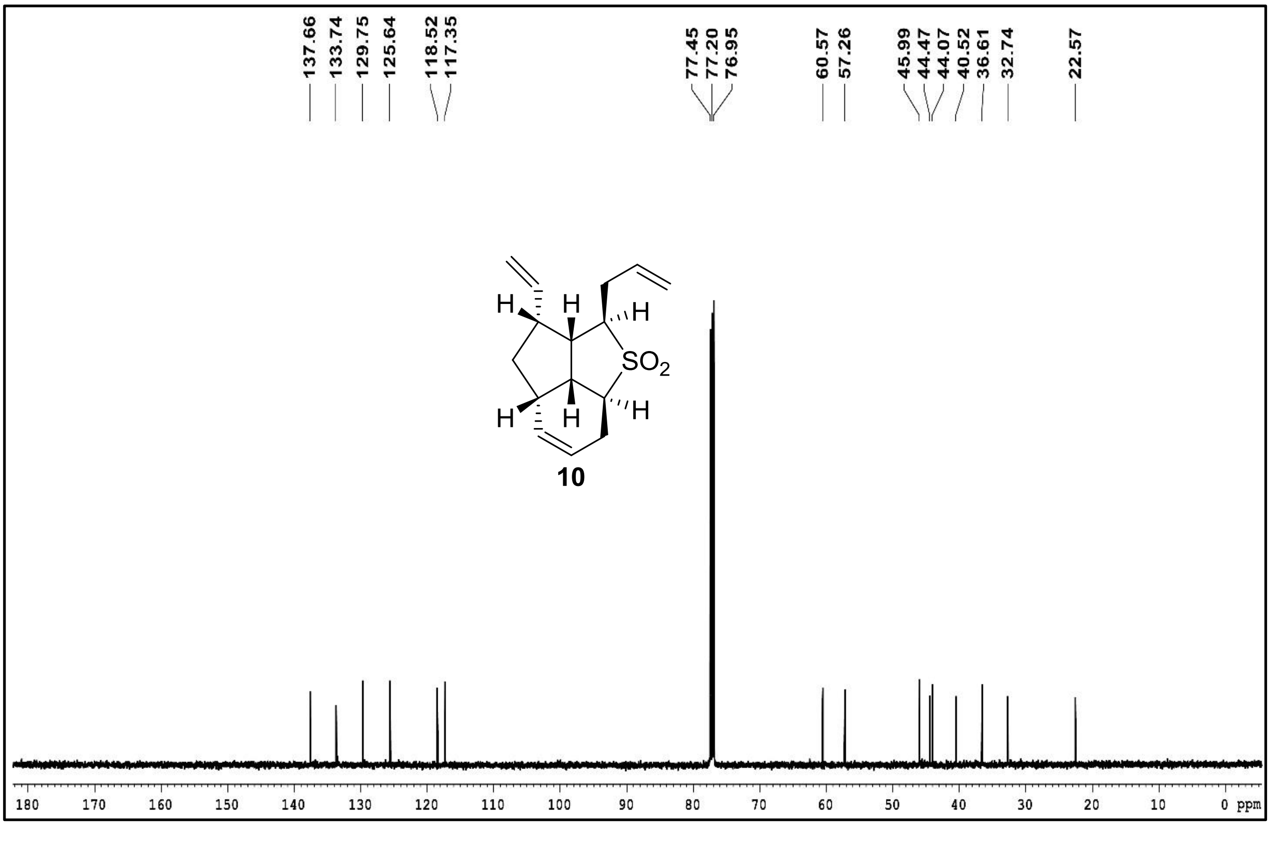

137.66
133.74
129.75
125.64
118.52
117.35
77.45
77.20
76.95
60.57
57.26
45.99
44.47
44.07
40.52
36.61
32.74
22.57
H
H
H
SO2
H
H
H
10
180
170
160
150
140
130
120
110
100
90
80
70
60
50
40
30
20
10
0 ppm

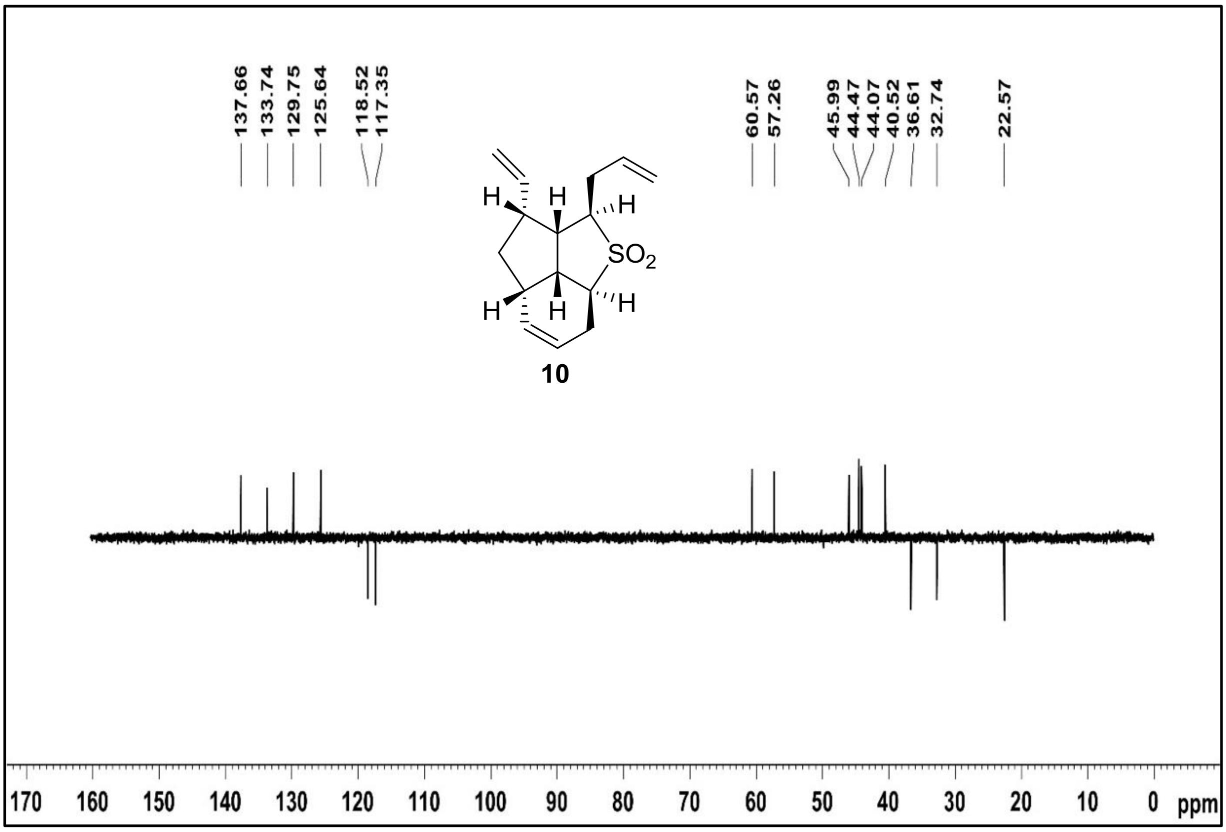

Acquisition Parameter

Source Type	ESI	Ion Polarity	Positive	Set Nebulizer	0.3 Bar
Focus	Active	Set Capillary	3800 V	Set Dry Heater	180 °C
Scan Begin	50 m/z	Set End Plate Offset	-500 V	Set Dry Gas	4.0 l/min
Scan End	1000 m/z	Set Collision Cell RF	1500.0 Vpp	Set Divert Valve	Source

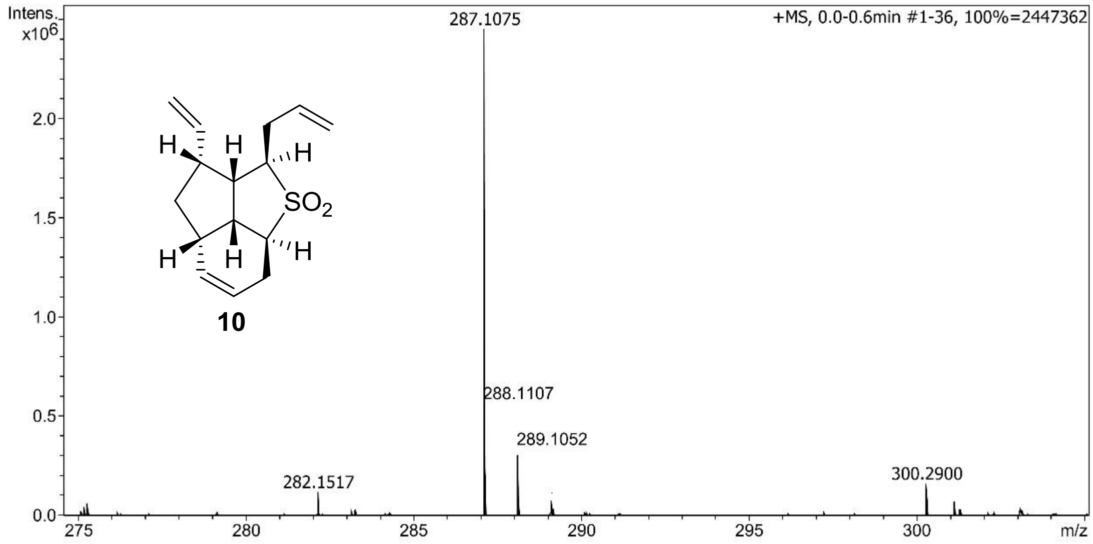

Meas. m/z	#	Ion Formula	m/z	err [ppm]	mSigma	# Sigma	Score	rdb	e⁻ Conf	N-Rule
287.1075	1	C15H20NaO2S	287.1076	0.4	30.0	1	100.00	5.5	even	ok

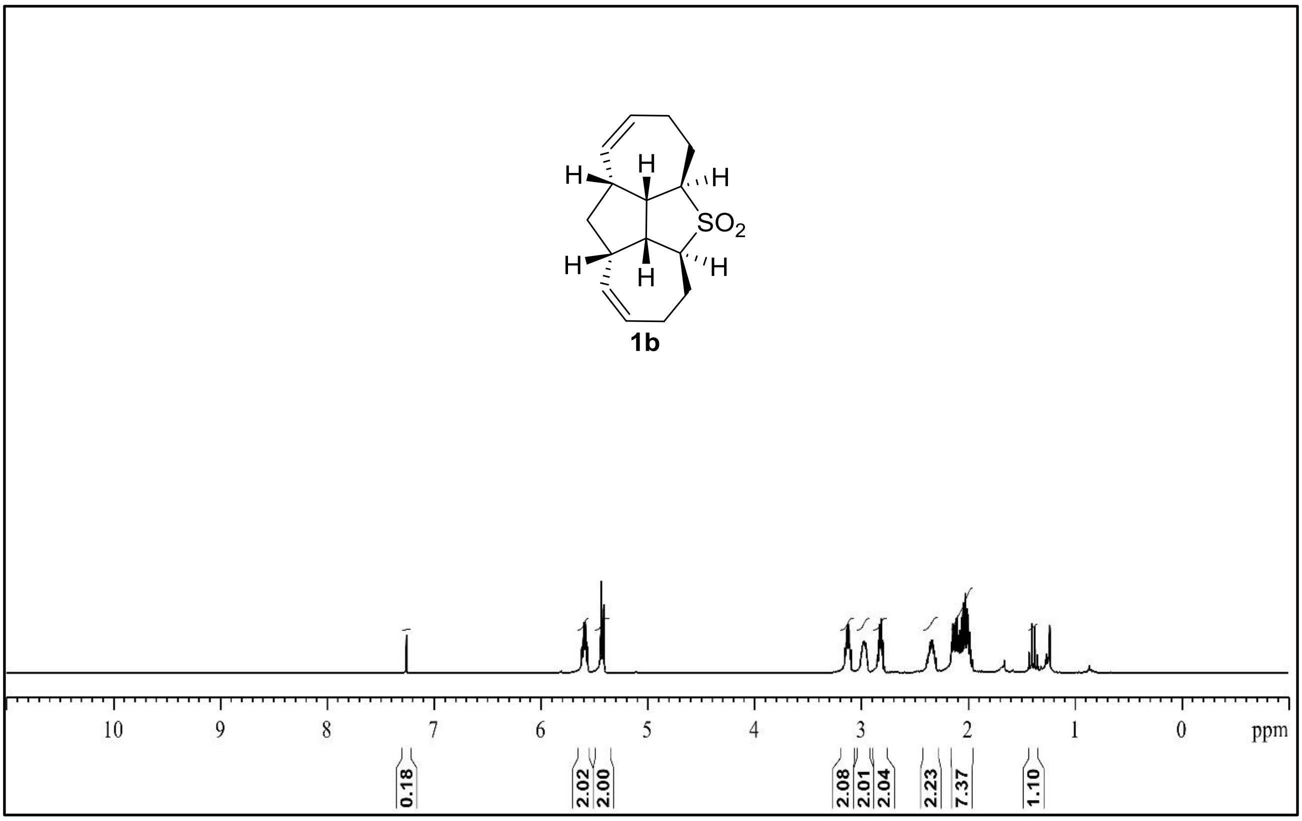
SO2
1b
10 9 8 7 6 5 4 3 2 1 0 ppm
0.18
2.02
2.00
2.08
2.01
2.04
2.23
7.37
1.10

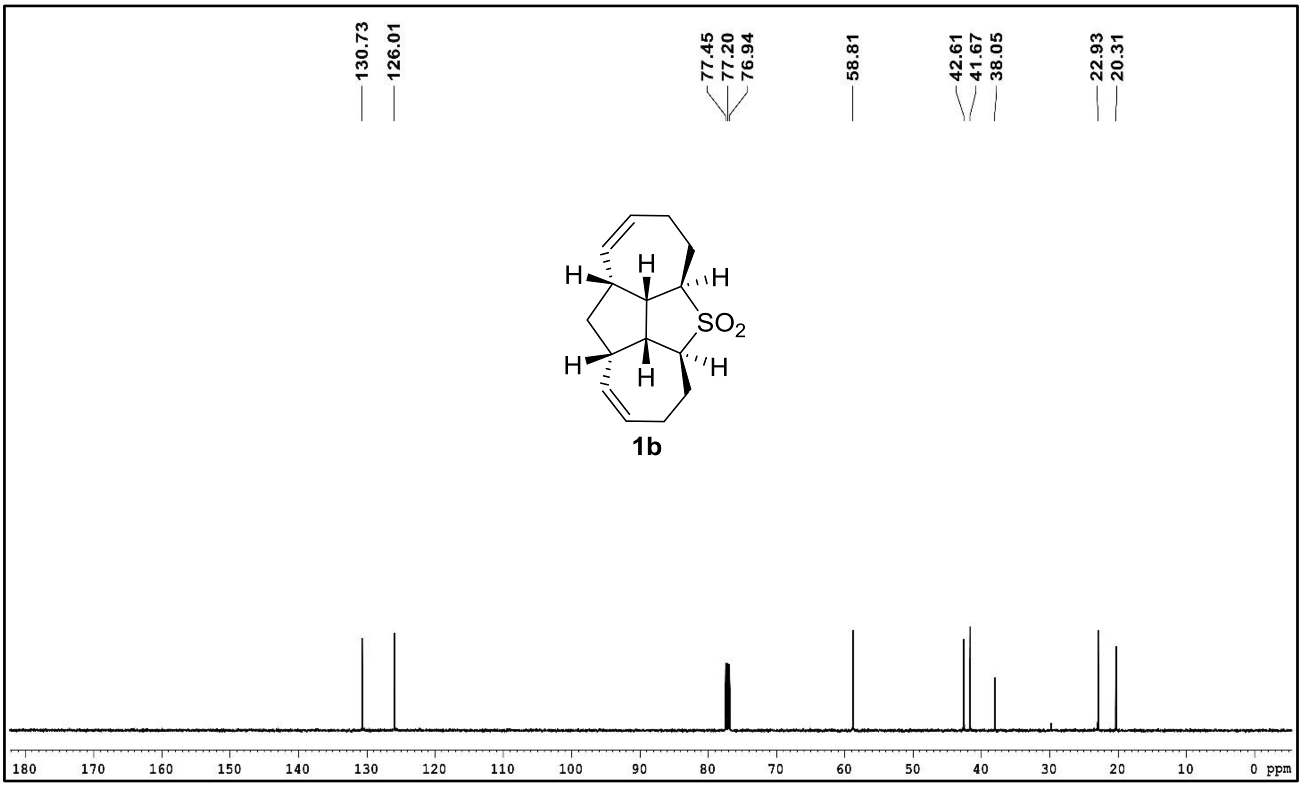
130.73
126.01
77.45
77.20
76.94
58.81
42.61
41.67
38.05
22.93
20.31
SO2
1b
180 170 160 150 140 130 120 110 100 90 80 70 60 50 40 30 20 10 0 ppm

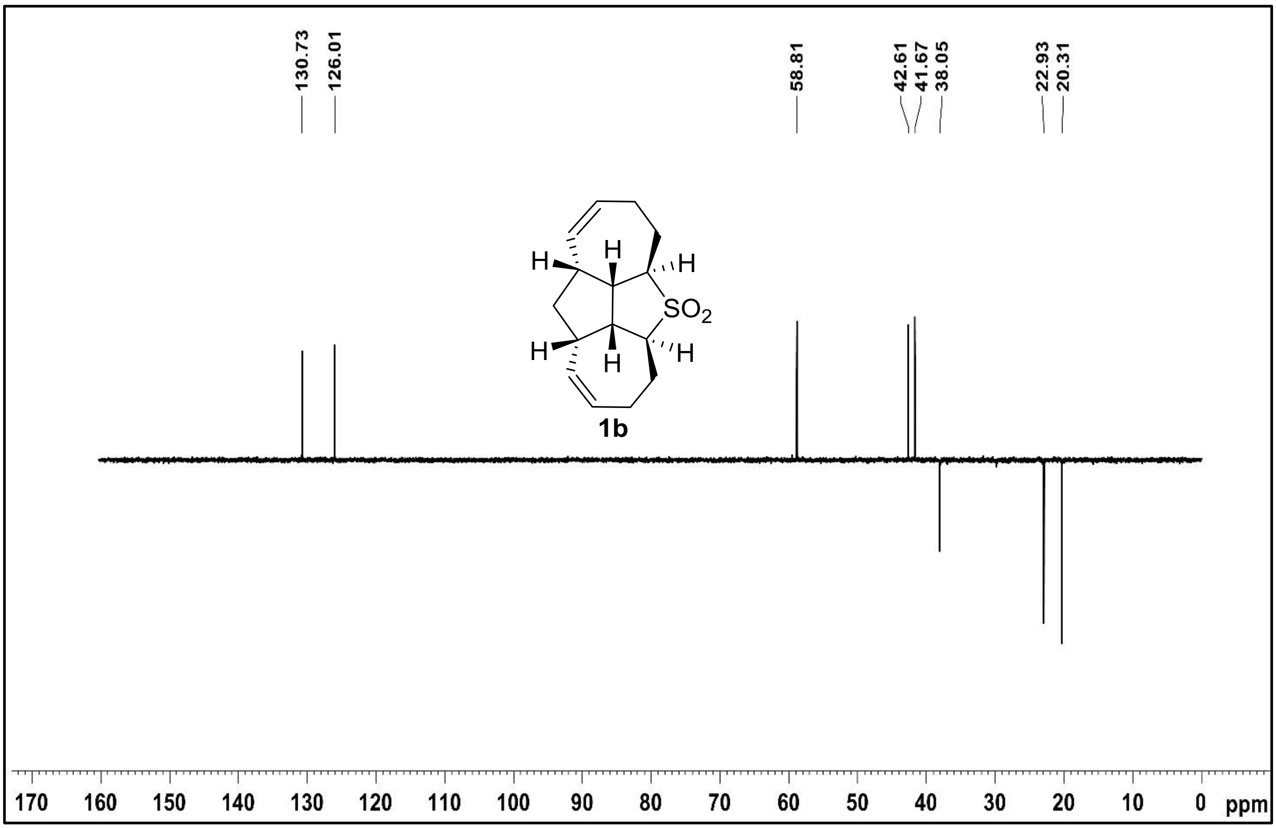

Acquisition Parameter

Source Type	ESI	Ion Polarity	Positive	Set Nebulizer	0.3 Bar
Focus	Active	Set Capillary	3800 V	Set Dry Heater	180 °C
Scan Begin	50 m/z	Set End Plate Offset	-500 V	Set Dry Gas	4.0 l/min
Scan End	1000 m/z	Set Collision Cell RF	1500.0 Vpp	Set Divert Valve	Source

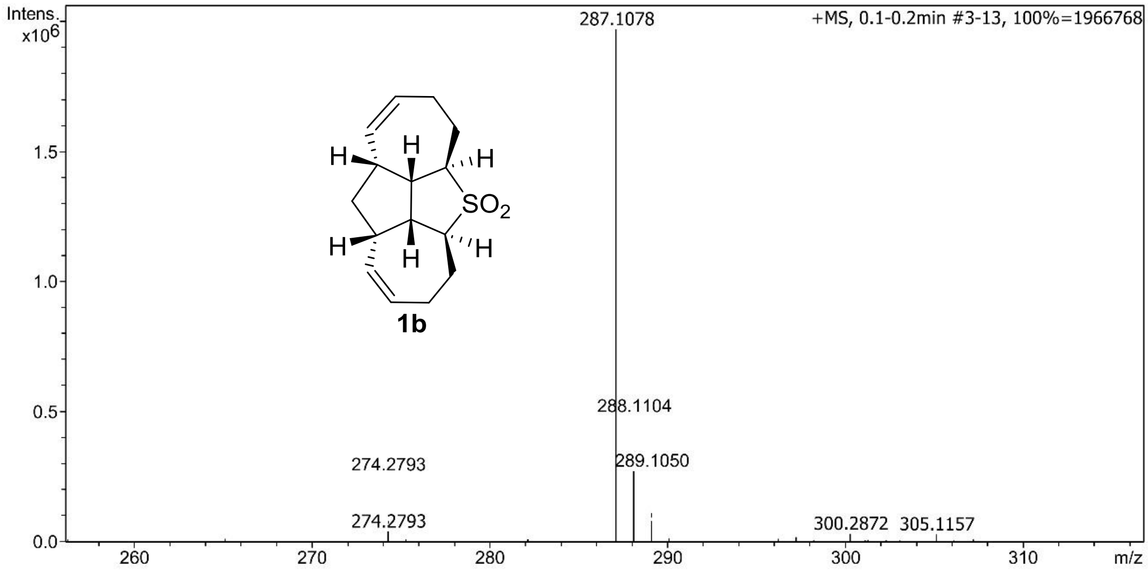

Meas. m/z	#	Ion Formula	m/z	err [ppm]	mSigma	# Sigma	Score	rdb	e⁻ Conf	N-Rule
287.1078	1	C15H20NaO2S	287.1076	-0.8	21.2	1	100.00	5.5	even	ok

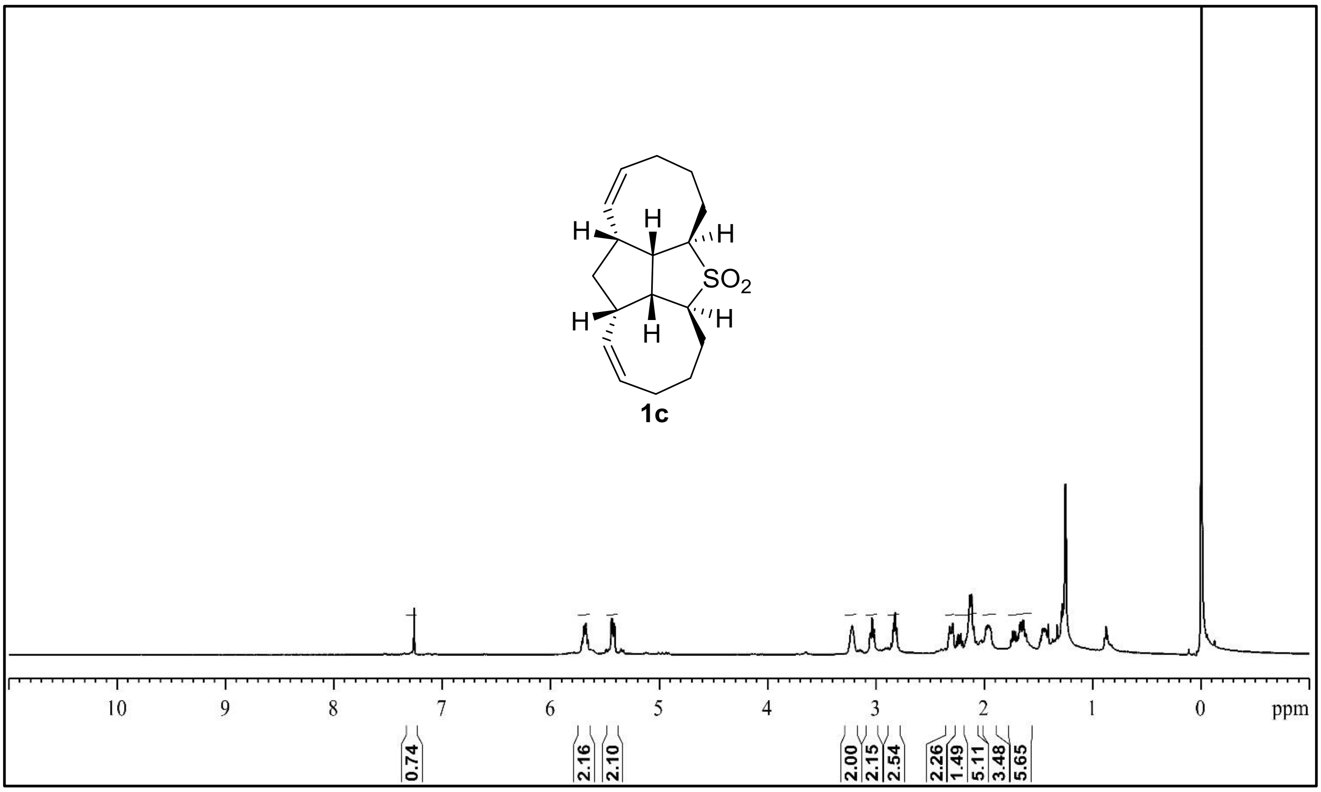
H
H
H
SO2
H
H
H
H
1c
10
9
8
7
6
5
4
3
2
1
0
ppm
0.74
2.16
2.10
2.00
2.15
2.54
2.26
1.49
5.11
3.48
5.65

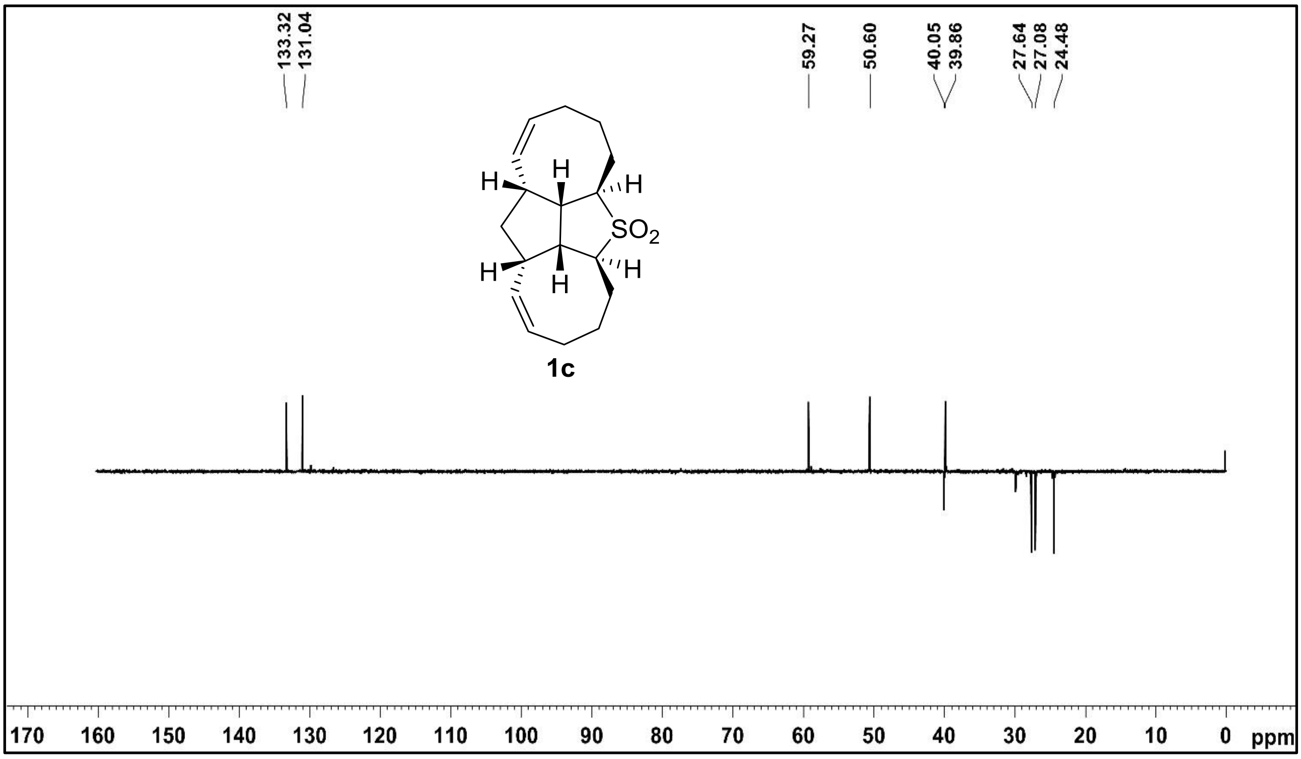
133.32
131.04
59.27
50.60
40.05
39.86
27.64
27.08
24.48
H
H
H
SO2
H
H
H
H
1c
170
160
150
140
130
120
110
100
90
80
70
60
50
40
30
20
10
0
ppm

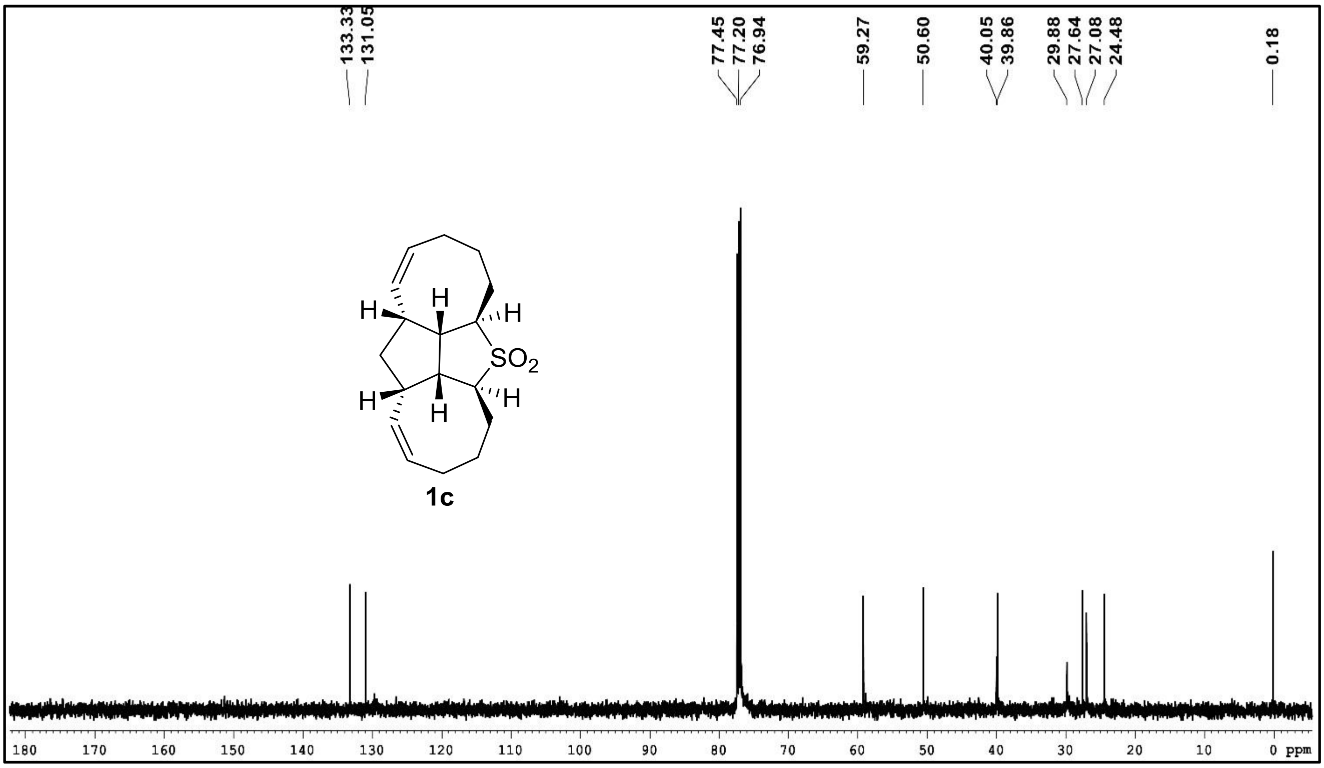

Acquisition Parameter

Source Type	ESI	Ion Polarity	Positive	Set Nebulizer	0.3 Bar
Focus	Active	Set Capillary	3800 V	Set Dry Heater	180 °C
Scan Begin	50 m/z	Set End Plate Offset	-500 V	Set Dry Gas	4.0 l/min
Scan End	1000 m/z	Set Collision Cell RF	1500.0 Vpp	Set Divert Valve	Source

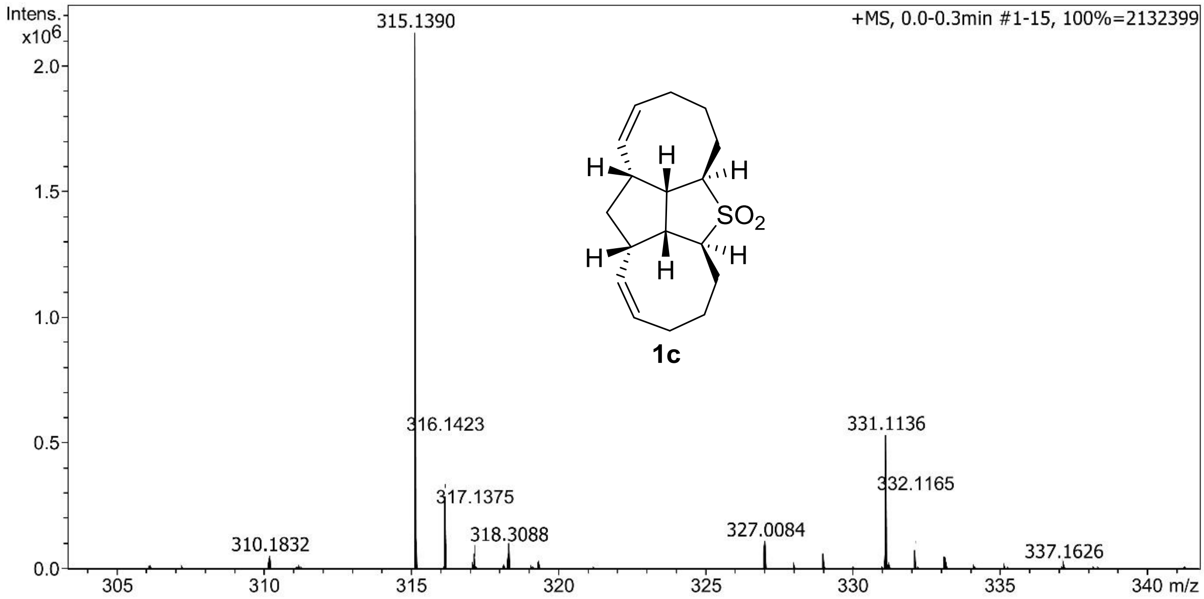

Meas. m/z	#	Ion Formula	m/z	err [ppm]	mSigma	# Sigma	Score	rdb	e⁻ Conf	N-Rule
315.1390	1	C17H24NaO2S	315.1389	0.3	35.7	1	100.00	5.5	even	ok

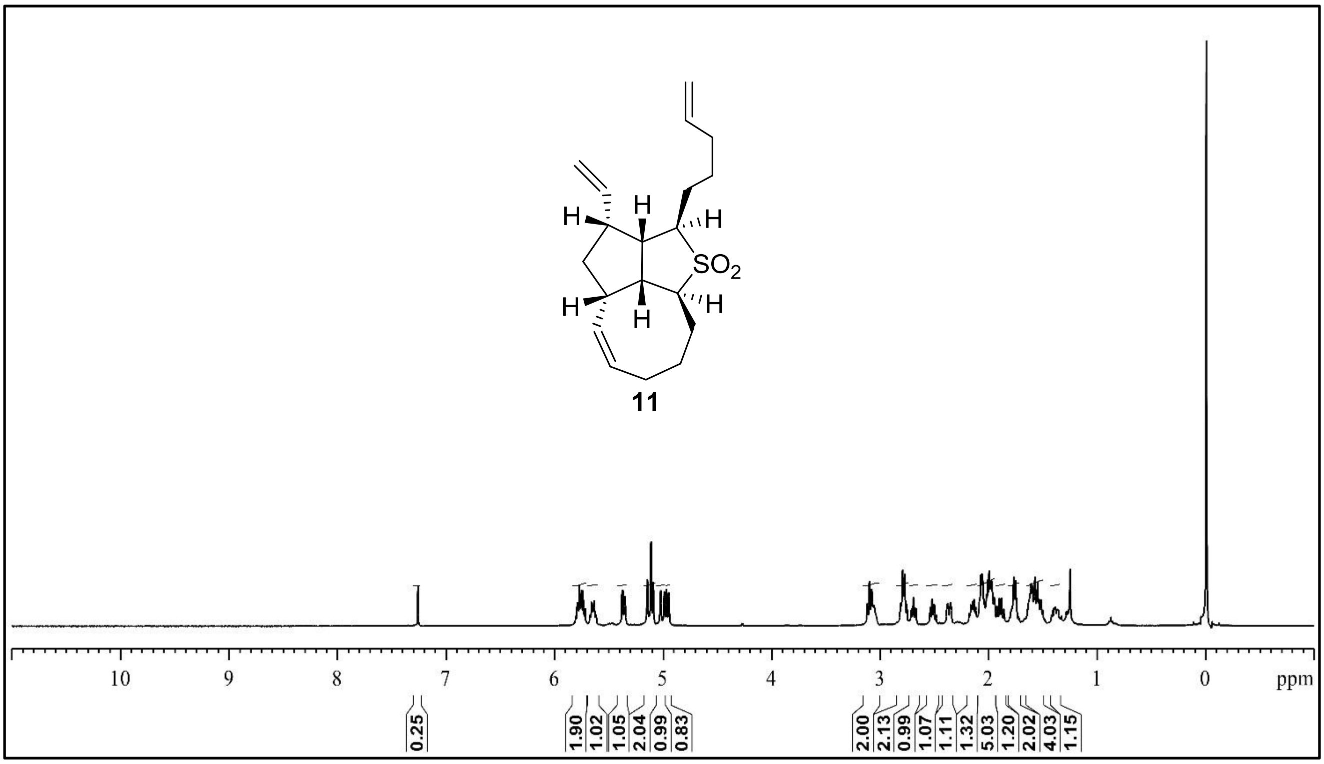
SO2
11
10
9
8
7
6
5
4
3
2
1
0
ppm
0.25
1.90
1.02
1.05
2.04
0.99
0.83
2.00
2.13
0.99
1.07
1.11
1.32
5.03
1.20
2.02
4.03
1.15

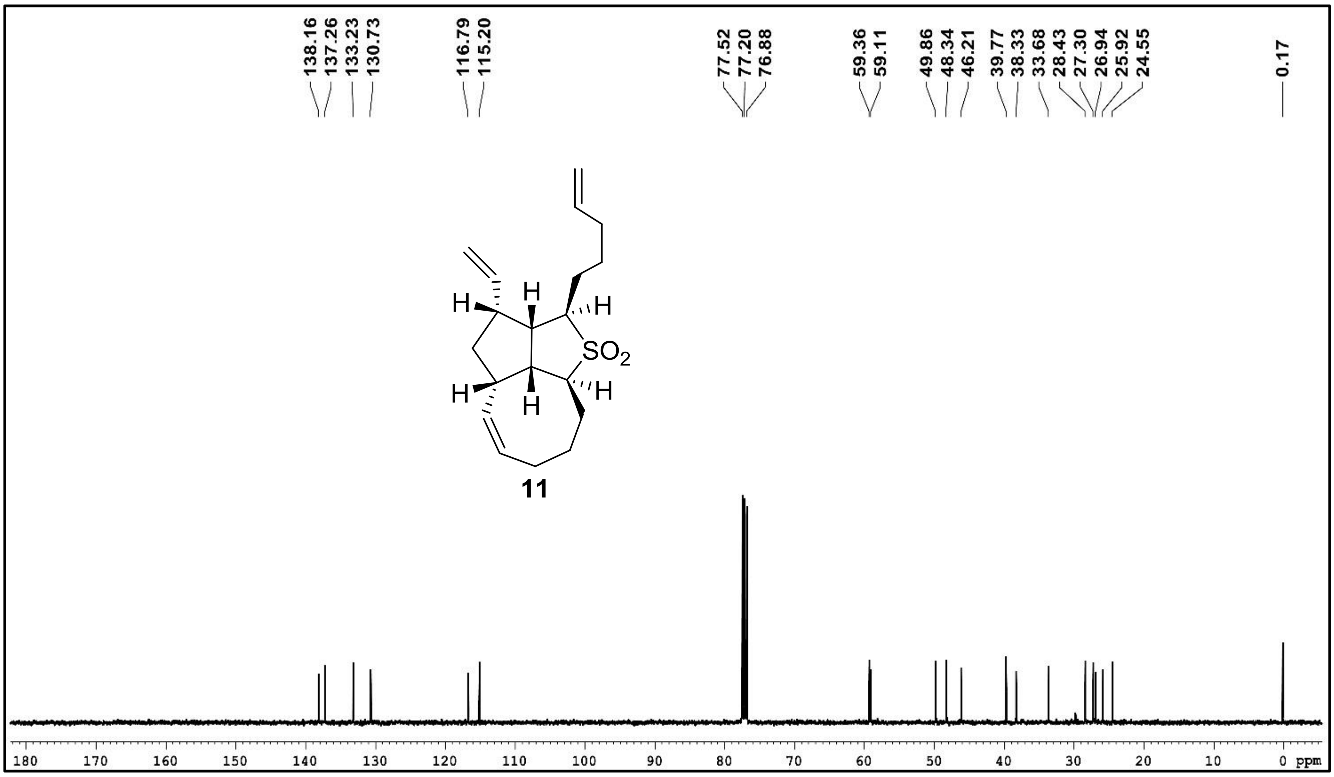
138.16
137.26
133.23
130.73
116.79
115.20
77.52
77.20
76.88
59.36
59.11
49.86
48.34
46.21
39.77
38.33
33.68
28.43
27.30
26.94
25.92
24.55
0.17
SO2
11
180
170
160
150
140
130
120
110
100
90
80
70
60
50
40
30
20
10
0 ppm

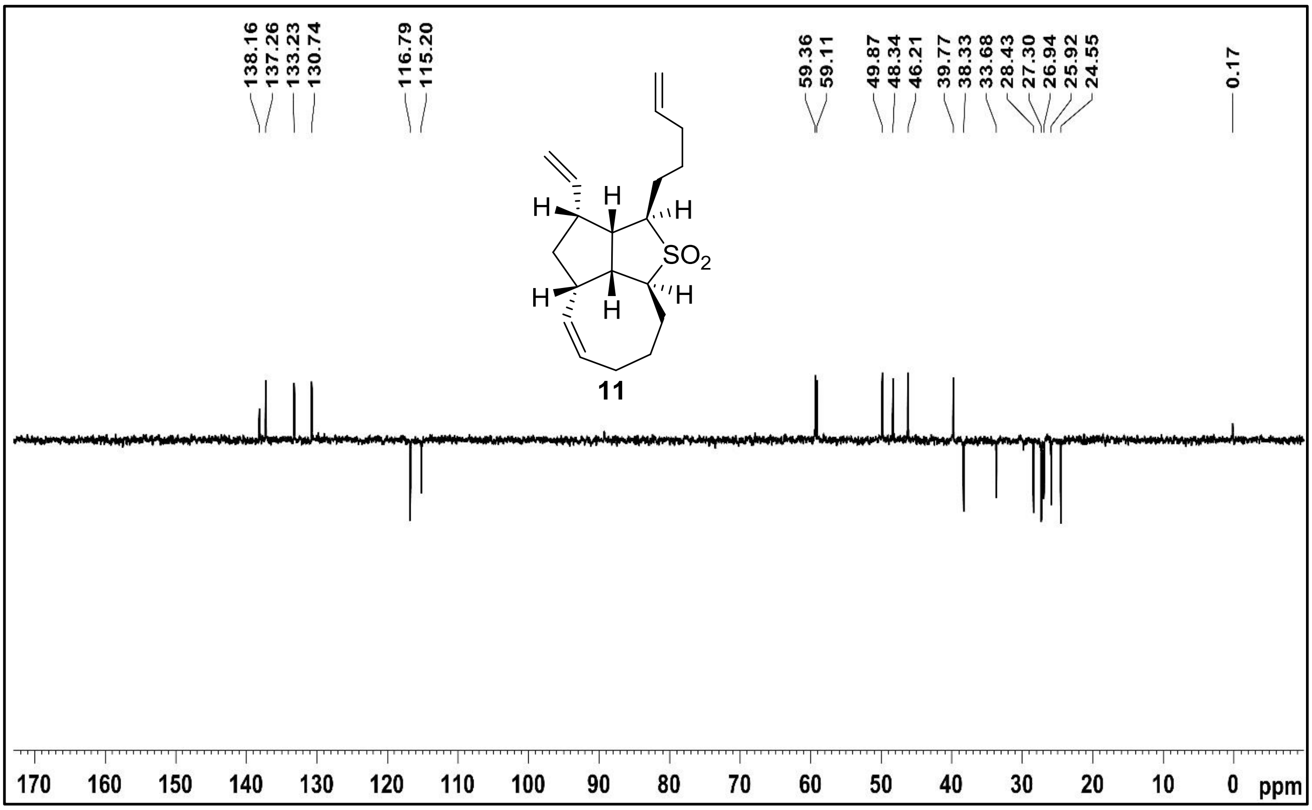

Acquisition Parameter

Source Type	ESI	Ion Polarity	Positive	Set Nebulizer	0.3 Bar
Focus	Active	Set Capillary	3800 V	Set Dry Heater	180 °C
Scan Begin	50 m/z	Set End Plate Offset	-500 V	Set Dry Gas	4.0 l/min
Scan End	1000 m/z	Set Collision Cell RF	1500.0 Vpp	Set Divert Valve	Source

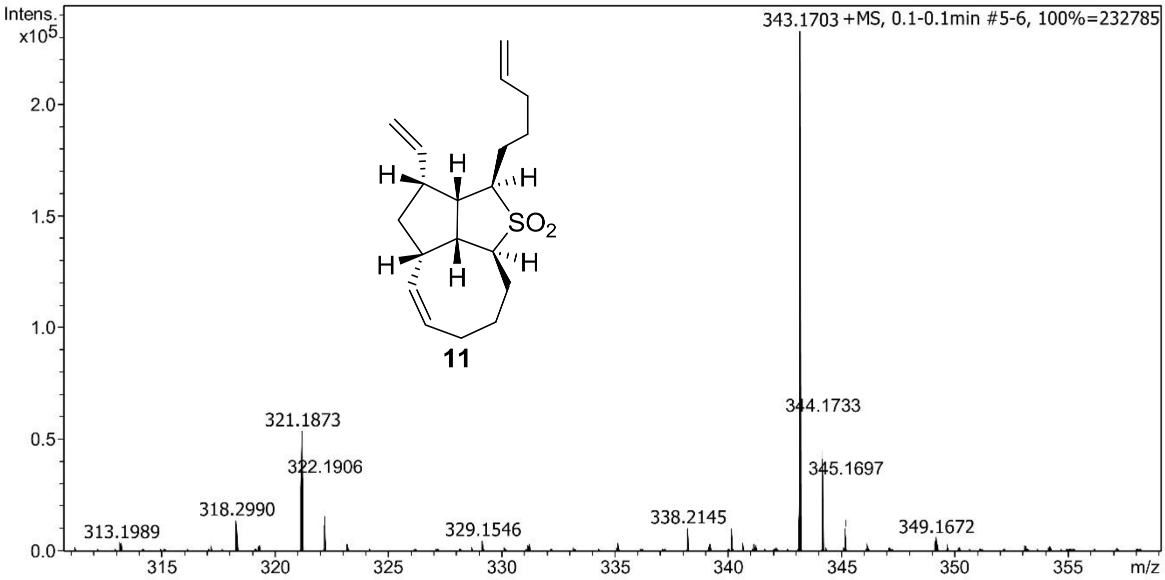

Meas. m/z	#	Ion Formula	m/z	err [ppm]	mSigma	# Sigma	Score	rdb	e⁻ Conf	N-Rule
343.1703	1	C19H28NaO2S	343.1702	0.2	24.8	1	100.00	5.5	even	ok

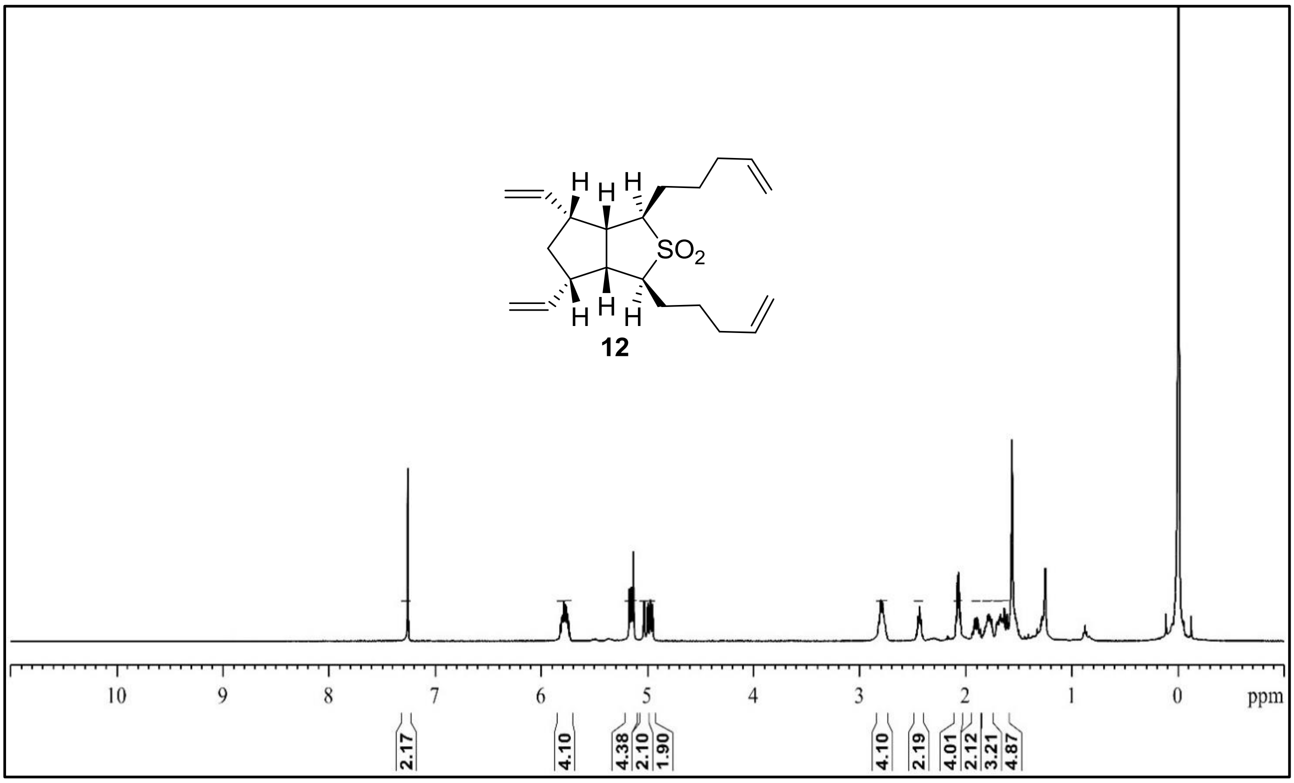
SO2
12
10
9
8
7
6
5
4
3
2
1
0
ppm
2.17
4.10
4.38
2.10
1.90
4.10
2.19
4.01
2.12
3.21
4.87

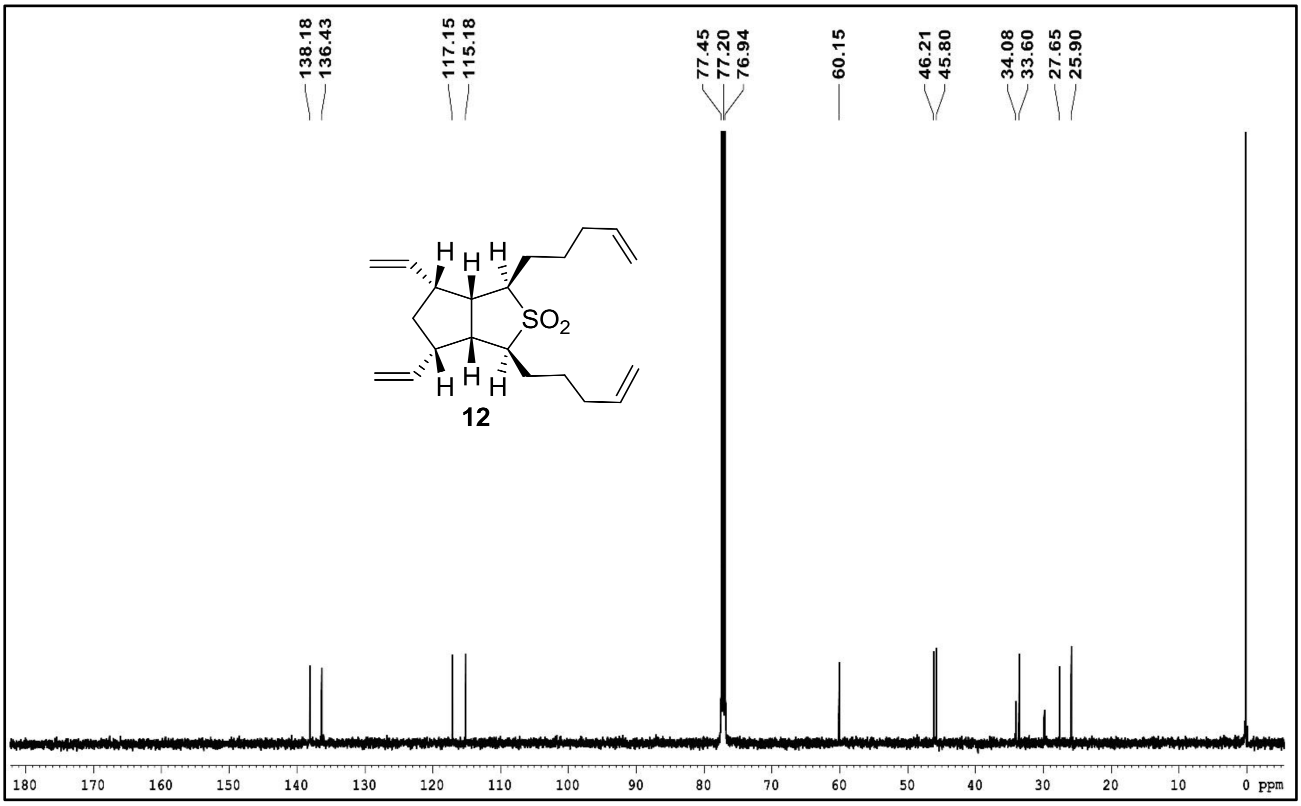
138.18
136.43
117.15
115.18
77.45
77.20
76.94
60.15
46.21
45.80
34.08
33.60
27.65
25.90
SO2
12
180
170
160
150
140
130
120
110
100
90
80
70
60
50
40
30
20
10
0 ppm

Acquisition Parameter

Source Type	ESI	Ion Polarity	Positive	Set Nebulizer	0.3 Bar
Focus	Active	Set Capillary	3800 V	Set Dry Heater	180 °C
Scan Begin	50 m/z	Set End Plate Offset	-500 V	Set Dry Gas	4.0 l/min
Scan End	1000 m/z	Set Collision Cell RF	1500.0 Vpp	Set Divert Valve	Source

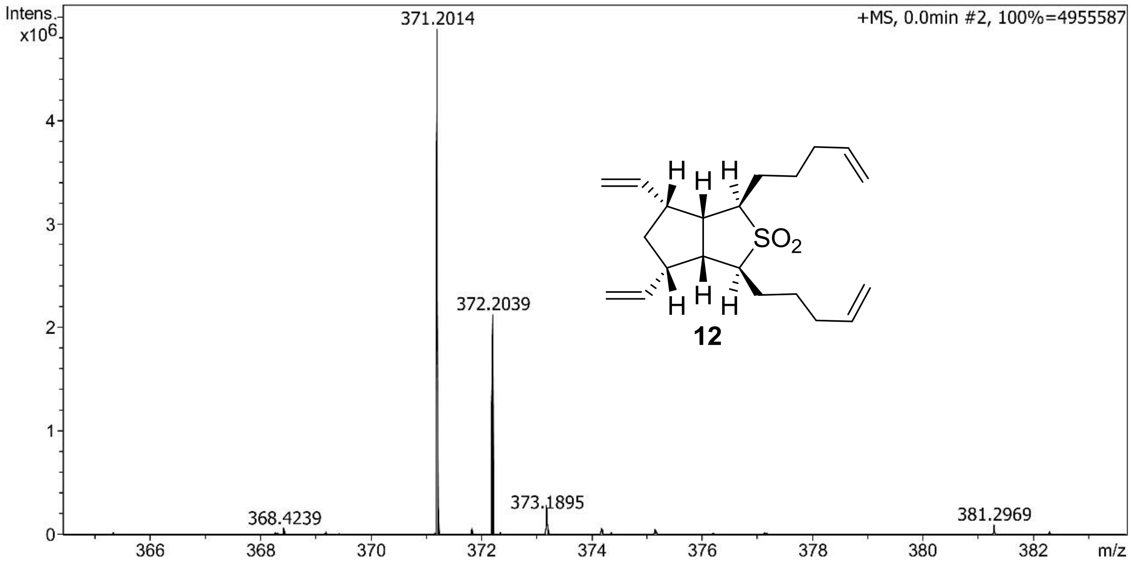

Meas. m/z	#	Ion Formula	m/z	err [ppm]	mSigma	# Sigma	Score	rdb	e⁻ Conf	N-Rule
371.2014	1	C21H32NaO2S	371.2015	0.2	99.3	1	100.00	5.5	even	ok

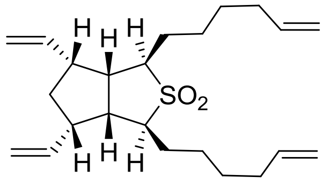

13

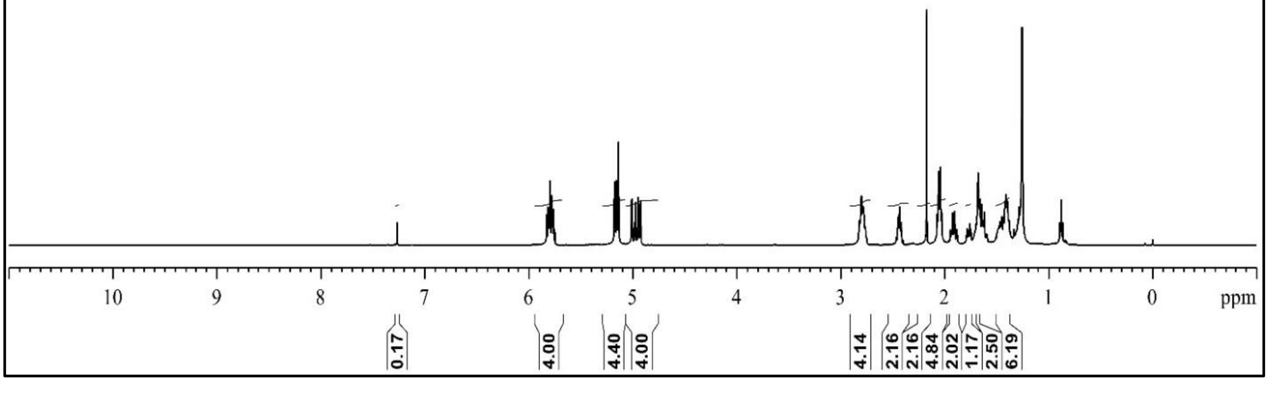

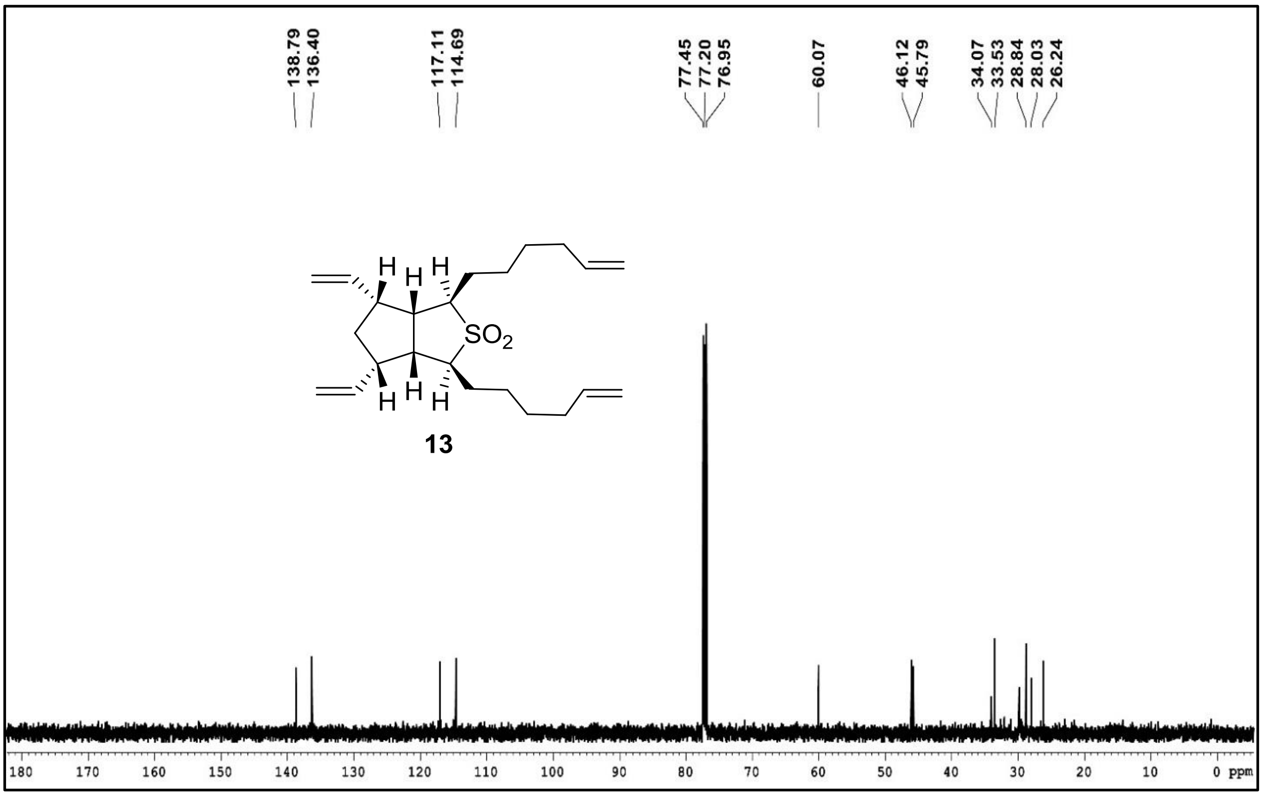

Acquisition Parameter

Source Type	ESI	Ion Polarity	Positive	Set Nebulizer	0.3 Bar
Focus	Active	Set Capillary	3700 V	Set Dry Heater	180 °C
Scan Begin	50 m/z	Set End Plate Offset	-500 V	Set Dry Gas	4.0 l/min
Scan End	500 m/z	Set Collision Cell RF	120.0 Vpp	Set Divert Valve	Source

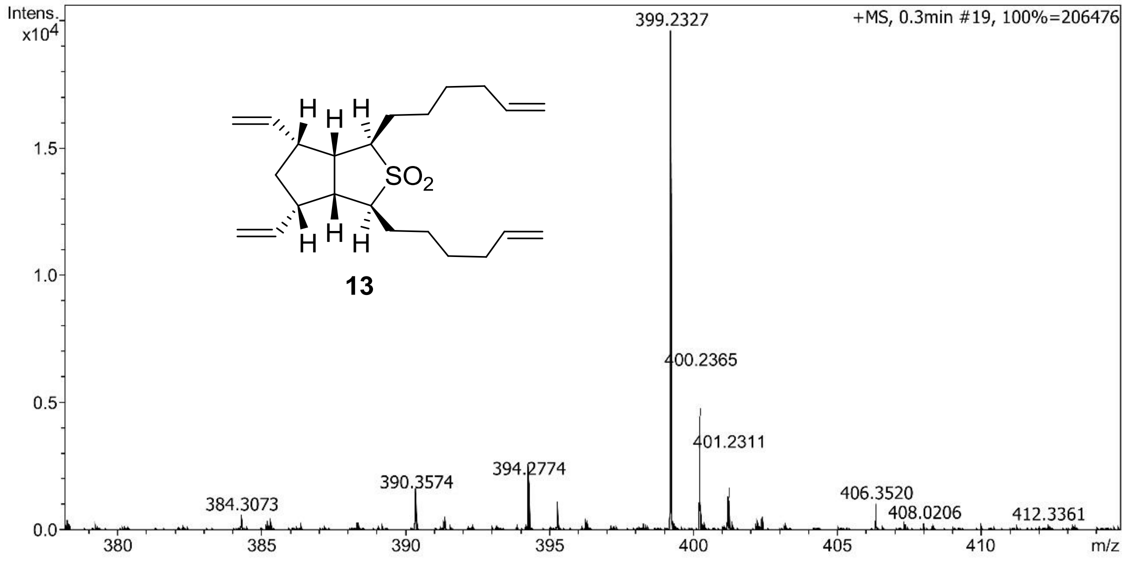

Meas. m/z	#	Ion Formula	m/z	err [ppm]	mSigma	# Sigma	Score	rdb	e⁻ Conf	N-Rule
399.2327	1	C23H36NaO2S	399.2328	-0.4	18.3	1	100.00	5.5	even	ok
